# Optimal Chemotherapy Scheduling for Chronic Lymphocytic Leukemia Under Immune and Allergy Constraints

**DOI:** 10.3390/e28080921

**Published:** 2026-08-17

**Authors:** Rawan Abdullah, Andrei Halanay, Lara Abou Orm

**Affiliations:** 1 Department of Arts and Sciences, Rafik Hariri University, P.O. Box 10, Damour-Chouf 2010, Lebanon; abouormla@rhu.edu.lb; 2 Department of Mathematics and Informatics, Faculty of Applied Sciences, National University of Science and Technology POLITEHNICA Bucharest, 060042 Bucharest, Romania; andrei.halanay@upb.ro

**Keywords:** optimal control, delay differential equations, chronic lymphocytic leukemia, chemotherapy scheduling, Pontryagin’s Maximum Principle, forward–backward sweep method, Kullback–Leibler divergence, Shannon entropy, Th1/Th2 balance, sensitivity analysis

## Abstract

We study an optimal control framework for chemotherapy administration in patients with chronic lymphocytic leukemia (CLL) while accounting for immune regulation and treatment-induced allergic reactions. The analysis is based on a previously developed nonlinear delay differential equation model describing the interactions between leukemic cells, immune populations, antigen-presenting cells, and cytokine dynamics, with three distinct biological delays. The chemotherapy infusion rate is introduced as a time-dependent control variable and optimized to reduce leukemic burden, shift the helper T-cell balance toward a Th1-dominant configuration associated with lower hypersensitivity risk, and preserve immune competence. Existence of an optimal control is established for arbitrary delays and horizon, without the commensurability hypothesis required by reductions in delay systems to higher-dimensional delay-free ones; the argument uses only that the control enters the dynamics affinely and the running cost concavely. Necessary optimality conditions are derived via Pontryagin’s Maximum Principle for systems with delays, and the resulting eleven-dimensional adjoint system, which carries advanced arguments generated by the three delays, is written out explicitly. A contraction estimate for the associated sweep operator yields both uniqueness of the optimal control on a short horizon and geometric convergence of the numerical scheme. The optimality system is solved by a forward–backward sweep adapted to the delayed setting, with documented convergence and grid independence and sensitivity analysis over kinetic parameters, delays, initial conditions and objective weights. The optimized schedule is compared not only with the untreated case and a low constant dose, but also with a constant infusion delivering the same cumulative exposure, so that the reported benefit is attributable to the temporal distribution of the dose rather than to its total amount. At equal exposure, the optimal schedule reaches each therapeutic milestone earlier—Th1 dominance 0.9 days sooner and a 90% leukemic reduction 1.6 days sooner—and attains a terminal leukemic burden lower by a factor of 2.25; a constant infusion of the same total dose reaches a comparable configuration later. The benefit of adaptive scheduling in this model is therefore principally one of rate of response at fixed drug exposure. We emphasize that the absolute Th2 population is not reduced by treatment; the reduction in hypersensitivity risk arises from the resulting Th1-dominant *relative* balance rather than from direct Th2 suppression. To characterize the therapeutic outcome in information-theoretic terms, we describe the two competing goals as distributional balances: an *allergy axis*, given by the Th1/Th2/Treg distribution, and a *leukemia axis*, given by the immune/leukemic distribution, each measured by its Shannon entropy and its Kullback–Leibler divergence to a healthy reference profile. These quantities are used in two roles. As diagnostics, they are evaluated along the computed trajectories, and the ordering of dosing strategies is shown to be robust across twenty alternative reference profiles. As an objective, the combined divergence is then taken as the running cost of a second optimal control problem; because it depends on the leukemic population only through a normalized fraction, it prescribes a markedly gentler schedule that administers 37% of the drug and still achieves a 93% leukemic reduction, against 98% for the population-based formulation. These results suggest that adaptive, immune-aware chemotherapy scheduling may accelerate disease control at fixed drug exposure, and that information-theoretic objectives offer a scale-free alternative formulation of the therapeutic goal.

## 1. Introduction

Cancer has long been a major focus of scientific research, and mathematical modeling continues to provide valuable tools for understanding disease progression and improving treatment strategies. In particular, dynamical models can assist in evaluating therapeutic protocols, optimizing drug administration, and balancing treatment efficacy against adverse side effects.

Chronic lymphocytic leukemia (CLL) is a hematological malignancy characterized by the abnormal accumulation of dysfunctional mature B lymphocytes. Although several therapies are available, chemotherapy remains an important treatment option in many clinical settings. However, treatment outcomes may be complicated by toxicity, immune suppression, tumor lysis syndrome (TLS), and drug-induced hypersensitivity reactions.

The authors of [1] investigated the cytotoxic effects of several chemotherapy agents, including chlorambucil, melphalan, and cytarabine, against leukemic cells. Their findings motivated mathematical formulations in which tumor growth and death rates depend explicitly on drug concentration.

A serious complication during CLL treatment is tumor lysis syndrome, which may arise when a large number of leukemic cells are destroyed rapidly, leading to dangerous metabolic imbalances. Clinical studies such as [2,3] highlight the need for carefully designed treatment schedules that control leukemic burden while avoiding excessive toxicity. That the schedule itself, and not only the agent or the total dose, influences outcome is supported by clinical experience: a retrospective comparison of alternative chlorambucil administration schedules in previously untreated CLL reported differences in response and tolerability between regimens [4]. The present study addresses the same question in a modeling framework, by treating the infusion profile as a decision variable and comparing schedules at fixed cumulative exposure.

In addition, hypersensitivity reactions to chlorambucil have been reported in [5,6], and cytokine release has been implicated in the infusion-related reactions observed when chlorambucil is combined with monoclonal antibody therapy [7]. From an immunological viewpoint, allergic responses are strongly linked to the balance between Th1 and Th2 helper T-cell populations, together with the regulatory role of Treg cells and cytokine signaling pathways; interleukin-6 in particular has been shown to play non-redundant roles in allergic inflammation [8].

Mathematical models in [9,10] demonstrated that a shift toward Th2 dominance may promote allergic inflammation. Motivated by these biological mechanisms, our previous studies developed delay differential equation models describing CLL treatment under desensitization protocols and analyzed several qualitative properties of the resulting systems; see [11,12,13,14].

In particular, the model presented in [13] established positivity, boundedness, global existence, and stability-related properties for a delayed CLL–immune interaction system under prescribed chemotherapy administration. These results provide the mathematical basis for the present work.

Optimal control has become a standard tool for the design of anticancer treatment schedules, and it is useful to situate the present contribution within that literature. Classical formulations optimize cytotoxic dosing against tumor burden and toxicity in ordinary differential equation models, and a recurrent qualitative finding is that schedules of bang–bang or bang-then-taper type arise whenever the control enters the dynamics linearly and the penalty on the control is quadratic. Extensions to delayed dynamics are comparatively recent: Gollmann and Maurer [15] developed the theory and numerical treatment of optimal control problems with multiple constant time delays in both state and control, and Rodrigues, Silva and Torres [16] applied delayed optimal control to an HIV model, obtaining necessary conditions of the same structure as those used here. On the numerical side, McAsey, Mou and Han [17] established convergence of the forward–backward sweep method under a Lipschitz-and-horizon-length condition, and Saleem et al. [18] surveyed its practical implementation. Relative to this body of work, the present paper differs in three respects. First, the controlled dynamics form an eleven-dimensional delay system with three distinct delays in which the control acts on a pharmacokinetic compartment rather than directly on the tumor equation, so that the drug influences the leukemic and immune populations only indirectly. Second, the objective couples disease control to an immunological side-effect constraint—the Th1/Th2/Treg balance governing hypersensitivity—rather than to a generic toxicity term. Third, the therapeutic goal is formulated in two ways, once in terms of cell populations and once in information-theoretic terms, and the two resulting optimal schedules are compared.

The aim of this paper is different from the previous desensitization-based approach. Here, we seek an *optimal time-dependent chemotherapy strategy* by introducing the drug infusion rate as a control variable. The objective is to determine dosing schedules that simultaneously:

- Reduce leukemic burden.
- Shift the helper T-cell balance toward a Th1-dominant configuration, which is associated with reduced allergy-related hypersensitivity risk.
- Preserve essential immune cell populations.
- Avoid excessive treatment exposure.

To achieve this goal, we formulate an optimal control problem governed by a nonlinear delay differential equation system. The existence of an optimal control is established for arbitrary delays and horizons; the argument requires only that the control enter the dynamics affinely and the running cost concavely, and therefore dispenses with the commensurability hypothesis on which reductions in delay systems to higher-dimensional delay-free systems rely. Necessary optimality conditions are derived using Pontryagin’s Maximum Principle for systems with delays, and the resulting eleven-dimensional adjoint system, which carries advanced arguments generated by the three delays, is written out explicitly. A contraction estimate for the associated sweep operator yields both uniqueness of the optimal control on a short horizon and geometric convergence of the numerical scheme.

The optimality system is solved numerically using a forward–backward sweep procedure adapted to the delayed setting. A methodological point governs how the results are reported. Since the computed optimal schedule delivers substantially more drug than a low constant infusion, comparing the two conflates the effect of dose *timing* with that of dose *amount*. We therefore include a constant infusion calibrated to deliver exactly the cumulative exposure of the optimal schedule, and it is against this exposure-matched protocol that the contribution of adaptive scheduling is assessed. The simulations show that at equal cumulative exposure the optimal schedule reaches every therapeutic milestone earlier—Th1 dominance, a fifty and a ninety percent leukemic reduction, and the minimum of the combined therapeutic divergence—while attaining a lower terminal leukemic burden. The benefit of adaptive dosing in this model is therefore principally one of rate of response at fixed drug exposure rather than of eventual position. The computed schedule does, however, administer the maximal dose over an initial phase of approximately three days, which stands in tension with the concern about rapid leukemic destruction noted above; this is discussed among the limitations.

Beyond population-level trajectories, the therapeutic goal of the treatment is itself naturally described in information-theoretic terms. The treatment must balance two competing objectives, each of which can be expressed as a distributional balance between cell populations. The first is an *allergy axis*: the relative proportions of Th1, Th2, and regulatory T cells determine the hypersensitivity risk, since a Th1-dominant configuration is associated with a low allergic response, whereas a shift toward Th2 dominance promotes allergic inflammation. The second is a *leukemia axis*: the relative proportions of immune effector and leukemic cells determine disease control, an immune-dominant configuration corresponding to effective tumor suppression. Each balance forms a probability distribution whose Shannon entropy measures its diversity and regulatory order, while the Kullback–Leibler divergence to a healthy reference profile (Th1-favorable on the allergy axis, immune-dominant on the leukemia axis) quantifies how far the current state lies from the desired one.

These information-theoretic quantities are used in two distinct roles, and it is worth separating them at the outset. In the first they are *diagnostics*: the optimal control is determined by the objective functional of Section 4, which is expressed directly in terms of cell populations, and the entropy and relative entropy are then evaluated *along* the resulting trajectories, providing a dimensionless summary comparable across strategies and across the two axes. In the second, developed in Section 6.7, the combined divergence itself becomes the running cost of a distinct optimal control problem, so that the information-theoretic quantity is the object being optimized rather than a measure of the outcome. Comparing the two resulting schedules answers a control-theoretic question rather than a descriptive one: whether the population-level and information-theoretic formulations of the therapeutic goal induce the same optimal policy. They do not. Because the divergence depends on the leukemic population only through a normalized fraction, it becomes insensitive to further absolute reduction once that fraction is small, and the divergence-driven schedule consequently administers substantially less drug while forgoing only a small part of the leukemic reduction achieved by the population-based schedule.

The main contributions of this paper are as follows:

1. Formulation of a delay optimal control framework for chemotherapy dosing in CLL with immune and allergy constraints, with an explicitly weighted objective functional.
2. Proof of existence of an optimal treatment strategy for arbitrary delays and horizons, without the commensurability hypothesis used in earlier reductions in delayed control systems, together with uniqueness, Lipschitz regularity and absence of singular arcs for the optimal schedule.
3. Derivation of necessary optimality conditions via Pontryagin’s Maximum Principle, including the explicit eleven-dimensional adjoint system with its advanced arguments, and a contraction estimate that simultaneously yields uniqueness of the optimizer and convergence of the forward–backward sweep.
4. Numerical solution by a forward–backward sweep adapted to the delayed setting, with documented convergence, grid independence, and sensitivity analysis over kinetic parameters, delays, initial conditions and objective weights.
5. Comparison of the optimized schedule against untreated, low-dose and *exposure-matched* constant protocols, isolating the effect of dose timing from that of cumulative dose.
6. Information-theoretic characterization of both the allergy axis (Th1/Th2/Treg) and the leukemia axis (immune/leukemic distribution) via the Shannon entropy and Kullback–Leibler divergence, in two roles: as diagnostics whose ordering of strategies is shown to be robust to the choice of reference profile, and as the running cost of a second optimal control problem whose solution is compared with the first.

## 2. Controlled Delay Model for CLL Dynamics

We consider the delayed mathematical model for chronic lymphocytic leukemia (CLL) treatment introduced in [13], where positivity, boundedness, global existence, and qualitative dynamical properties were previously investigated. In the present work, this framework is extended to an optimal control setting by introducing a time-dependent chemotherapy administration rate u(t), representing the normalized drug infusion intensity. The state equations are otherwise unchanged; the sole modification is that the infusion term in the pharmacokinetic equation, previously a prescribed function of time, is now a decision variable.

The model describes the interactions among leukemic cells, immune populations, antigen-presenting cells, cytokines, and the chemotherapeutic drug. In particular, it incorporates helper T-cell subpopulations (Th1, Th2), regulatory T cells, naive immune cells, activated antigen-presenting cells, immune effector cells, and living/dead leukemic cells.

Three biologically relevant delays are included:

- $\tau_{1}$: propagation delay associated with transport from the central to the peripheral compartment;
- $\tau_{2}$: delay corresponding to cytokine production and signaling;
- $\tau_{3}$: leukemic cell-cycle duration.

The control variable satisfies

$$u\left(t\right)\in \left[0,1\right],\;t\in \left[0,t_{f}\right],$$

where u(t)=0 corresponds to no treatment and u(t)=1 represents maximal normalized drug administration. Note that *u* enters the system only through the additive infusion term of the pharmacokinetic equation, so that the right-hand side is affine in the control; this structural fact is used in the existence proof of Section 4.

Let the state variables be defined as follows:

$$\begin{array}{cc} x_{1}: & \text{naive T-helper cells},\\ x_{2}: & \text{Th1 cells},\\ x_{3}: & \text{Th2 cells},\\ x_{4}: & \text{regulatory T cells},\\ x_{5}: & \text{naive APCs},\\ x_{6}: & \text{mature APCs},\\ x_{7}: & \text{drug concentration},\\ x_{8}: & \text{cytokine concentration},\\ x_{9}: & \text{living leukemic cells},\\ x_{10}: & \text{dead leukemic cells},\\ x_{11}: & \text{immune effector cells}. \end{array}$$

All concentrations are expressed per microlitre, as set out in Section 8.

The controlled delay differential system is therefore written as

(1) $$\left\{\begin{array}{c} {\dot{x}}_{1}=\alpha -\beta_{1}x_{1}-x_{1}x_{6}\left(t-\tau_{1}\right)\frac{x_{2}}{1+\mu_{2}x_{3}}-\phi x_{1}x_{6}\left(t-\tau_{1}\right)x_{3}-\kappa x_{1}x_{6}\left(t-\tau_{1}\right)x_{4}\\ {\dot{x}}_{2}=-\beta_{2}x_{2}+v\frac{x_{1}x_{6}\left(t-\tau_{1}\right)x_{2}}{\left(1+\mu_{r}x_{4}\right)\left(1+\mu_{2}x_{3}\right)}\\ {\dot{x}}_{3}=-\beta_{3}x_{3}+\phi v\frac{x_{1}x_{6}\left(t-\tau_{1}\right)x_{3}\left(1+\mu_{2}x_{3}\right)}{\left(1+\mu_{r}x_{4}\right)\left(1+\mu_{1}x_{2}+\mu_{2}x_{3}\right)}\\ {\dot{x}}_{4}=-\beta_{4}x_{4}+\kappa vx_{1}x_{6}\left(t-\tau_{1}\right)x_{4}-\eta_{r}\frac{x_{8}x_{4}}{1+x_{8}}\\ {\dot{x}}_{5}=a_{APC}-\beta_{0}x_{7}x_{5}-\gamma_{11}x_{5}\\ {\dot{x}}_{6}=\beta_{0}x_{7}x_{5}-\gamma_{12}x_{6}-\mu_{0}x_{6}x_{4}\\ {\dot{x}}_{7}=u\left(t\right)-\gamma_{D}x_{7}-\frac{\mu_{D}x_{9}x_{7}}{a+x_{7}}\\ {\dot{x}}_{8}=-\gamma_{2}x_{8}+k_{1}\left[x_{1}\left(t-\tau_{2}\right)+x_{2}\left(t-\tau_{2}\right)+x_{3}\left(t-\tau_{2}\right)+x_{4}\left(t-\tau_{2}\right)+x_{6}\left(t-\tau_{2}\right)\right]\\ {\dot{x}}_{9}=-\gamma_{L}x_{9}-\Psi \left(x_{9}\right)+2e^{-\gamma_{L}\tau_{3}}\Psi \left(x_{9}\left(t-\tau_{3}\right)\right)-c_{1}x_{9}x_{11}-\frac{\mu_{L}x_{9}x_{7}}{a+x_{7}}\\ {\dot{x}}_{10}=\gamma_{L}x_{9}-dx_{10}+\frac{\mu_{L}x_{9}x_{7}}{a+x_{7}}\\ {\dot{x}}_{11}=s-mx_{11}+\frac{\rho x_{9}x_{11}}{\gamma +x_{9}}-\frac{\delta x_{11}x_{7}}{c+x_{7}}-c_{2}x_{9}x_{11} \end{array}\right.$$

The leukemic self-renewal flux is taken as

(2) $$\Psi \left(x\right)=\beta_{L}\;\frac{\theta^{2}x^{2}}{\theta^{2}+x^{2}},$$

a Hill-type function of the leukemic load with half-saturation θ. The factor $2e^{-\gamma_{L}\tau_{3}}$ accounts for the doubling of cells completing the cycle, discounted by mortality over its duration $\tau_{3}$. The corresponding per-cell renewal rate is $\Psi \left(x\right)/x=\beta_{L}\theta^{2}x/\left(\theta^{2}+x^{2}\right)$, which increases with leukemic density for x<θ and decreases beyond it, attaining its maximum at x=θ. This density-dependent form differs from the monotonically decreasing per-cell rate of [19]; the leukemic burdens attained in the present simulations remain well below θ, so the model operates throughout in the regime where the flux is approximately $\beta_{L}x^{2}$, as discussed in Section 6.2 and in the limitations.

Initial data are prescribed through history functions on the interval

$$\left[-\tau_{\max},0\right],\;\tau_{\max}=\max\left\{\tau_{1},\tau_{2},\tau_{3}\right\},$$

namely,

$$x_{i}\left(t\right)=\varphi_{i}\left(t\right),\;t\in \left[-\tau_{\max},0\right],\;i=1,\ldots ,11.$$

The qualitative properties of the uncontrolled dynamics and the biological relevance of the model parameters were established in [13]. In the present study, the main novelty lies in treating u(t) as a decision variable and determining an optimal chemotherapy schedule that balances treatment efficacy, immune preservation, and reduction in allergy-related adverse responses. For the general theory of delay differential systems underlying the analysis that follows—existence, continuation and stability of solutions of functional differential equations—we refer to [20,21].

## 3. Well-Posedness and Preliminary Properties

The optimal control analysis developed below relies on three structural properties of delayed System (1): existence and uniqueness of solutions for every admissible control, invariance of the nonnegative orthant, and boundedness of the state on the finite horizon. These were established in [13] for the uncontrolled dynamics with a prescribed infusion profile; we restate them in the form required here, since the control now ranges over an entire admissible class rather than being a fixed function of time.

Throughout, admissible controls are the measurable functions

$$U=\left\{u:\left[0,t_{f}\right]\to \left[0,1\right]\;:\;u\;\mathrm{measurable}\right\},$$

and we write $\tau_{\max}=\max\left\{\tau_{1},\tau_{2},\tau_{3}\right\}$ and

$$C^{+}=\left\{\varphi \in C\left(\left[-\tau_{\max},0\right];R^{11}\right)\;:\;\varphi_{i}\left(s\right)\geq 0\;\;\forall s,\;i=1,\ldots ,11\right\}$$

for the cone of nonnegative continuous initial histories.

**Proposition 1** (Well-posedness of the controlled system)**.** *Let $\varphi \in C^{+}$ and let u∈U. Then, the following apply:*

*(i)*  (Local existence and uniqueness.) *The right-hand side F of *(1)* is continuous in t and locally Lipschitz in $\left(x,x_{\tau }\right)$ on $R_{+}^{11}\times R_{+}^{11\times 3}$, uniformly for u∈[0,1]. Consequently, Equation *(1)* admits a unique solution on a maximal interval $\left[0,t_{\max}\right)$, obtained by the method of steps on successive intervals of length $\min\{\tau_{1},\tau_{2},\tau_{3}\}$.*
*(ii)*  (Positivity.) *$x_{i}\left(t\right)\geq 0$ for all $t\in [0,t_{\max})$ and all i. Each equation of Equation *(1)* has the form ${\dot{x}}_{i}=P_{i}-x_{i}Q_{i}$ with $P_{i}\geq 0$ and $Q_{i}$ bounded on bounded sets whenever the state is nonnegative, so that*
  $$x_{i}\left(t\right)=\left[x_{i}\left(0\right)+\int_{0}^{t}P_{i}\left(r\right)e^{\int_{0}^{r}Q_{i}}\;dr\right]e^{-\int_{0}^{t}Q_{i}}\;\geq \;0.$$
*(iii)*  (Boundedness and global existence.) *On every finite horizon $\left[0,t_{f}\right]$ there is a constant $K=K(\varphi ,t_{f})$, independent of u∈U, with ${∥x\left(t\right)∥}_{\infty }\leq K$ for all $t\in [0,t_{f}]$. In particular $t_{\max}>t_{f}$.*

**Sketch.**  Parts (i) and (ii) are as in ([13], Section 3); the control does not affect them, entering only additively and boundedly in the $x_{7}$ equation. For (iii) we chain a priori estimates, each uniform in *u*. From ${\dot{x}}_{7}\leq 1-\gamma_{D}x_{7}$ we get $x_{7}\left(t\right)\leq \max\left\{x_{7}\left(0\right),1/\gamma_{D}\right\}$, uniform because u≤1. Then ${\dot{x}}_{5}\leq a_{APC}-\gamma_{11}x_{5}$ and ${\dot{x}}_{6}\leq \beta_{0}x_{7}x_{5}-\gamma_{12}x_{6}$ bound the antigen-presenting compartments. Self-renewal flux Equation (2) is bounded above by $\beta_{L}\theta^{2}$, since $\Psi \left(x\right)\leq \beta_{L}\theta^{2}$ for every x≥0; the $x_{9}$ equation therefore gives ${\dot{x}}_{9}\leq 2e^{-\gamma_{L}\tau_{3}}\beta_{L}\theta^{2}$, so that $x_{9}$ grows at most linearly on $\left[0,t_{f}\right]$, and the same bounds $x_{10}$ and, through the linear supply term, $x_{11}$. Finally, $x_{1},\ldots ,x_{4}$ are bounded because their gain terms are dominated by $v\;x_{1}x_{6}\left(t-\tau_{1}\right)$ with $x_{6}$ already bounded, and $x_{8}$ by the linear cytokine equation driven by bounded arguments. □

**Remark** **1.** *The uniformity in u in part (iii) is what the existence argument of Theorem 1 consumes: it makes the family of state trajectories generated by U uniformly bounded and equicontinuous on $\left[0,t_{f}\right]$.*

## 4. Optimal Control Formulation

We formulate the chemotherapy scheduling problem for chronic lymphocytic leukemia (CLL) as a finite-horizon optimal control problem, where the chlorambucil infusion rate is treated as a time-dependent control variable. The objective is to determine a dosing strategy that balances therapeutic efficacy, immune preservation, and reduction in treatment-related adverse effects.

The controlled dynamics are governed by the delay differential system (Equation (1)), subject to the initial history conditions

$$x_{i}\left(t\right)=\varphi_{i}\left(t\right),\;t\in \left[-\tau_{\max},0\right],\;i=1,\ldots ,11.$$

Recall from Section 3 the admissible control set

$$U=\left\{u:\left[0,t_{f}\right]\to \left[0,1\right]\;|\;u\;\mathrm{is}\;\mathrm{measurable}\right\},$$

where u(t)=0 corresponds to no drug administration and u(t)=1 denotes the maximal normalized dose.

The therapeutic goals are as follows:

- Enhance anti-tumor immune activity through Th1 cells $x_{2}\left(t\right)$ and effector cells $x_{11}\left(t\right)$.
- Shift the helper T-cell balance toward Th1 dominance by penalizing the Th2 population $x_{3}\left(t\right)$.
- Reduce the leukemic cell population $x_{9}\left(t\right)$.
- Avoid excessive chemotherapy exposure.

Accordingly, we consider the weighted objective functional

(3) $$J\left(u\right)=\int_{0}^{t_{f}}\left[A_{1}x_{11}\left(t\right)+A_{2}x_{2}\left(t\right)-A_{3}x_{3}\left(t\right)-A_{4}x_{9}\left(t\right)-A_{5}u^{2}\left(t\right)\right]\;dt,\;A_{1},\ldots ,A_{5}>0.$$

The positive terms reward immune reinforcement, whereas the negative terms penalize leukemic burden, allergy-related responses, and excessive drug administration. The coefficients $A_{1},\ldots ,A_{4}$ are trade-off weights between the four therapeutic goals and $A_{5}$ sets the price of drug exposure relative to them. Throughout the numerical experiments the baseline choice is

$$A_{1}=A_{2}=A_{3}=A_{4}=A_{5}=1,$$

weighting the four population objectives equally; the dependence of the computed schedule on this choice is examined in Section 6.2.

**Remark 2** (Dimensional consistency)**.** *All state variables in Equation *(1)* are expressed in normalized concentration units, and u is a dimensionless normalized infusion intensity in [0,1]. The weights $A_{1},\ldots ,A_{4}$ therefore carry the reciprocal of the corresponding concentration unit and $A_{5}$ is dimensionless, so every term of the integrand of Equation *(3)* has the same physical dimension. The values quoted above are those of the weights in this normalized system of units. This is why the weights must be displayed rather than absorbed into the integrand: the value of J is meaningful only relative to a stated normalization, and comparisons of J across dosing strategies are made at fixed weights.*

The optimal control problem is to determine $u^{*}\in U$ such that

$$J\left(u^{*}\right)=\max_{u\in U}J\left(u\right).$$

To derive necessary optimality conditions, we apply Pontryagin’s Maximum Principle for systems with discrete delays [22,23]. This yields a coupled state–adjoint system together with a characterization of the optimal control.

The resulting optimality system is solved numerically by a forward–backward sweep iterative procedure adapted to incorporate delay terms in both the state and adjoint equations. This framework provides a practical methodology for designing adaptive chemotherapy schedules that improve treatment effectiveness while limiting adverse immune responses.

**Theorem 1** (Existence of an optimal control)**.** Let $\varphi \in C^{+}$ and let $t_{f}>0$ be arbitrary. Consider delayed System *(1)* with the objective functional Equation *(3)* over the admissible set U. Then there exists $u^{*}\in U$ with

$$J\left(u^{*}\right)=\max_{u\in U}J\left(u\right),$$

and the associated trajectory $x^{*}$ is the unique solution of Equation *(1)* corresponding to $u^{*}$. No arithmetic relation among the delays $\tau_{1},\tau_{2},\tau_{3}$ and the horizon $t_{f}$ is required.

**Proof.**  The admissible set is nonempty and *J* is bounded above on U, since by Proposition 1(iii) all admissible trajectories remain in a compact set $K\subset R_{+}^{11}$ depending only on φ and $t_{f}$; write $J^{*}:=\sup_{u\in U}J\left(u\right)<\infty$. Let $(u^{n})\subset U$ be a maximizing sequence with associated states $x^{n}$.

*Step 1 (compactness of controls).* U is a bounded, convex, weak-* closed subset of $L^{\infty }\left(0,t_{f}\right)$, hence weak-* sequentially compact by the Banach–Alaoglu theorem together with the separability of $L^{1}\left(0,t_{f}\right)$. Passing to a subsequence, $u^{n}{⇀}^{*}u^{*}$ with $u^{*}\in U$, the pointwise bounds being preserved because U is convex and weak-* closed.

*Step 2 (convergence of the states).* Since $x^{n}\left(t\right)\in K$ and $|u^{n}|\leq 1$, the right-hand side *F* is uniformly bounded along the sequence, so $\left({\dot{x}}^{n}\right)$ is uniformly bounded and $\left(x^{n}\right)$ is uniformly bounded and equicontinuous on $\left[0,t_{f}\right]$. By the Arzelà–Ascoli theorem a further subsequence converges uniformly, $x^{n}\to x$ in $C(\left[0,t_{f}\right];R^{11})$; the same convergence holds for the shifted arguments $x^{n}\left(\cdot -\tau_{j}\right)$, since the initial histories are fixed and uniform convergence is preserved under translation.

*Step 3 (admissibility of the limit).* Write Equation (1) in integral form. The control appears only in the seventh component and only additively:

$$x_{7}^{n}\left(t\right)=\varphi_{7}\left(0\right)+\int_{0}^{t}u^{n}\left(r\right)\;dr-\int_{0}^{t}\left[\gamma_{D}x_{7}^{n}+\frac{\mu_{D}x_{9}^{n}x_{7}^{n}}{a+x_{7}^{n}}\right]dr.$$

The first integral converges to $\int_{0}^{t}u^{*}$ for each *t* by weak-* convergence, since $\chi_{\left[0,t\right]}\in L^{1}$. The remaining integrals, and all components i≠7, involve *u* nowhere, and their integrands converge uniformly by Step 2 together with the local Lipschitz continuity of *F* on K. Hence *x* satisfies Equation (1) with control $u^{*}$ and history φ, and by Proposition 1(i) it is the unique such solution: $x=x^{*}$.

*Step 4 (upper semicontinuity of the objective).* Decompose

$$J\left(u^{n}\right)=\underset{=:J_{1}\left(x^{n}\right)}{\underset{︸}{\int_{0}^{t_{f}}\;\left[A_{1}x_{11}^{n}+A_{2}x_{2}^{n}-A_{3}x_{3}^{n}-A_{4}x_{9}^{n}\right]dt}}\;-\;A_{5}\;\int_{0}^{t_{f}}\;{\left(u^{n}\right)}^{2}dt.$$

The term $J_{1}$ is linear in the state and converges to $J_{1}\left(x^{*}\right)$ by uniform convergence. The map $u\mapsto \int_{0}^{t_{f}}u^{2}$ is convex and strongly continuous on $L^{2}\left(0,t_{f}\right)$, hence weakly lower semicontinuous, so

$$\int_{0}^{t_{f}}{\left(u^{*}\right)}^{2}dt\;\leq \;\underset{n\to \infty }{\mathrm{lim inf}}\int_{0}^{t_{f}}{\left(u^{n}\right)}^{2}dt.$$

Combining, $J\left(u^{*}\right)\geq {\mathrm{lim sup}}_{n\to \infty }J\left(u^{n}\right)=J^{*}$, and since $u^{*}\in U$ also $J\left(u^{*}\right)\leq J^{*}$. Therefore $J\left(u^{*}\right)=J^{*}$. □

**Remark 3** (On the role of the structural hypotheses)**.** *The proof uses two structural features of the problem and nothing else. First, F is* affine *in u: the control enters only as the additive infusion term of the pharmacokinetic equation. Second, the integrand of Equation *(3)* is* concave *in u, the control appearing only through $-A_{5}u^{2}$. Together these are precisely the convexity condition on the extended velocity set*

$$N\left(t,x,x_{\tau }\right)=\left\{\left(F(t,x,x_{\tau },u),\;z\right):\;z\leq A_{1}x_{11}+A_{2}x_{2}-A_{3}x_{3}-A_{4}x_{9}-A_{5}u^{2},\;u\in \left[0,1\right]\right\}$$

*required by the Filippov–Cesari theory [24]: for $u_{a},u_{b}\in \left[0,1\right]$ and μ∈[0,1], affineness of F gives $\mu F\left(u_{a}\right)+\left(1-\mu \right)F\left(u_{b}\right)=F\left(\mu u_{a}+\left(1-\mu \right)u_{b}\right)$, while concavity of the integrand gives the corresponding inequality in the last coordinate, so N is convex. If the control acted nonlinearly on the dynamics, or if the penalty on u were replaced by an $L^{1}$ term, this convexity could fail, and a relaxed formulation would be required.*

*Earlier treatments of delayed optimal control problems of this type obtain existence by exploiting rational dependence of the delays to transform the system into an equivalent delay-free system of higher dimension [25,26], which requires $\tau_{j}=k_{j}\tau$ and $t_{f}=L\tau$ with integer $k_{j},L$. The argument above shows that for the present problem that hypothesis is dispensable: the delays enter only through arguments handled by the Arzelà–Ascoli step, and the compactness required is supplied by the uniform bounds of Proposition 1(iii) rather than by a finite-dimensional reduction. The delays of Table 8 are consequently used exactly as tabulated, with no rounding to a commensurate grid.*

## 5. Adjoint System and Optimality Conditions

To derive the necessary conditions for optimality, we apply Pontryagin’s Maximum Principle for systems with discrete delays [22,23]. Let

$$x\left(t\right)={\left(x_{1}\left(t\right),\ldots ,x_{11}\left(t\right)\right)}^{⊤},$$

and denote the delayed state variables by

$$x_{\tau }\left(t\right)=\left(x\left(t-\tau_{1}\right),x\left(t-\tau_{2}\right),x\left(t-\tau_{3}\right)\right).$$

Controlled System (1) can be written in compact form as

$$\dot{x}\left(t\right)=F\left(t,x\left(t\right),x_{\tau }\left(t\right),u\left(t\right)\right).$$

We introduce the adjoint vector

$$\lambda \left(t\right)={\left(\lambda_{1}\left(t\right),\ldots ,\lambda_{11}\left(t\right)\right)}^{⊤},$$

and define the Hamiltonian by

(4) $$H\left(t,x,x_{\tau },u,\lambda \right)=A_{1}x_{11}+A_{2}x_{2}-A_{3}x_{3}-A_{4}x_{9}-A_{5}u^{2}+\lambda^{⊤}F\left(t,x,x_{\tau },u\right).$$

For an optimal control $u^{*}\left(t\right)$ with associated trajectory $x^{*}\left(t\right)$, there exists an adjoint function λ(t) satisfying

(5) $${\dot{\lambda }}_{i}\left(t\right)=-\left(\frac{\partial H}{\partial x_{i}}\left(t\right)+\sum_{j=1}^{3}\chi_{\left[0,t_{f}-\tau_{j}\right]}\left(t\right)\frac{\partial H}{\partial y_{i\tau_{j}}}\left(t+\tau_{j}\right)\right),\;i=1,\ldots ,11,$$

where $y_{i\tau_{j}}$ denotes the argument of the Hamiltonian corresponding to the delayed state $x_{i}\left(t-\tau_{j}\right)$, with terminal conditions

$$\lambda_{i}\left(t_{f}\right)=0,\;i=1,\ldots ,11.$$

We draw attention to the structure of Equation (5): the delays of the state system appear in the adjoint system as *advanced* arguments evaluated at $t+\tau_{j}$, which is what dictates both the explicit form given in Section 5.1 and the numerical scheme of Section 5.4.

The optimal control is characterized by the maximization condition

$$H\left(t,x^{*}\left(t\right),x_{\tau }^{*}\left(t\right),u^{*}\left(t\right),\lambda \left(t\right)\right)=\max_{0\leq u\leq 1}H\left(t,x^{*}\left(t\right),x_{\tau }^{*}\left(t\right),u,\lambda \left(t\right)\right),$$

which is made explicit in the characterization given in Section 5.3 below.

### 5.1. Explicit Form of the Adjoint System

Because the model is eleven-dimensional with three distinct delays, we display the adjoint system in full; this is also what is implemented in the backward sweep of Section 5.4. Abbreviate

$$y_{1}\left(t\right):=x_{6}\left(t-\tau_{1}\right),\;W:=1+\mu_{2}x_{3},\;R:=1+\mu_{r}x_{4},\;M:=1+\mu_{1}x_{2}+\mu_{2}x_{3},$$

$$G\left(z\right):=\Psi^{\prime }\left(z\right)=\frac{2\beta_{L}\theta^{4}z}{{\left(\theta^{2}+z^{2}\right)}^{2}},\;\chi_{j}\left(t\right):=\chi_{\left[0,\;t_{f}-\tau_{j}\right]}\left(t\right),\;j=1,2,3,$$

and write

(6) $$\Phi :=\lambda_{1}\;\left(\frac{x_{1}x_{2}}{W}+\phi \;x_{1}x_{3}+\kappa \;x_{1}x_{4}\right)-\frac{\lambda_{2}\;v\;x_{1}x_{2}}{WR}-\frac{\lambda_{3}\;\phi \;v\;x_{1}x_{3}\;W}{RM}-\kappa \;\lambda_{4}\;v\;x_{1}x_{4},$$

all quantities in Equation (6) being evaluated at the same instant. With Hamiltonian Equation (4), adjoint Equation (5) reads

(7) $$\begin{array}{cc} {\dot{\lambda }}_{1}=\; & \lambda_{1}\;\left(\beta_{1}+\phi x_{3}y_{1}+\kappa x_{4}y_{1}+\frac{x_{2}y_{1}}{W}\right)-\frac{\lambda_{2}vx_{2}y_{1}}{WR}-\frac{\lambda_{3}\phi vx_{3}y_{1}W}{RM}-\kappa \lambda_{4}vx_{4}y_{1}\;-\;\chi_{2}\;k_{1}\lambda_{8}\left(t+\tau_{2}\right), \end{array}$$

(8) $$\begin{array}{cc} {\dot{\lambda }}_{2}=\; & -A_{2}+\frac{\lambda_{1}x_{1}y_{1}}{W}+\lambda_{2}\;\left(\beta_{2}-\frac{vx_{1}y_{1}}{WR}\right)+\frac{\lambda_{3}\mu_{1}\phi vx_{1}x_{3}y_{1}W}{RM^{2}}\;-\;\chi_{2}\;k_{1}\lambda_{8}\left(t+\tau_{2}\right), \end{array}$$

(9) $$\begin{array}{cc} {\dot{\lambda }}_{3}=\; & A_{3}-\lambda_{1}\;\left(\frac{\mu_{2}x_{1}x_{2}y_{1}}{W^{2}}-\phi x_{1}y_{1}\right)+\frac{\lambda_{2}\mu_{2}vx_{1}x_{2}y_{1}}{W^{2}R}\\ \; & +\lambda_{3}\;\left(\beta_{3}+\frac{\mu_{2}\phi vx_{1}x_{3}y_{1}W}{RM^{2}}-\frac{\mu_{2}\phi vx_{1}x_{3}y_{1}}{RM}-\frac{\phi vx_{1}y_{1}W}{RM}\right)\;-\;\chi_{2}\;k_{1}\lambda_{8}\left(t+\tau_{2}\right), \end{array}$$

(10) $$\begin{array}{cc} {\dot{\lambda }}_{4}=\; & \kappa \lambda_{1}x_{1}y_{1}+\frac{\lambda_{2}\mu_{r}vx_{1}x_{2}y_{1}}{WR^{2}}+\frac{\lambda_{3}\mu_{r}\phi vx_{1}x_{3}y_{1}W}{R^{2}M}+\lambda_{4}\;\left(\beta_{4}+\frac{\eta_{r}x_{8}}{1+x_{8}}-\kappa vx_{1}y_{1}\right)+\lambda_{6}\mu_{0}x_{6}\\ \; & \;-\;\chi_{2}\;k_{1}\lambda_{8}\left(t+\tau_{2}\right), \end{array}$$

(11) $$\begin{array}{cc} {\dot{\lambda }}_{5}=\; & \lambda_{5}\left(\beta_{0}x_{7}+\gamma_{11}\right)-\lambda_{6}\beta_{0}x_{7}, \end{array}$$

(12) $$\begin{array}{cc} {\dot{\lambda }}_{6}=\; & \lambda_{6}\left(\gamma_{12}+\mu_{0}x_{4}\right)\;+\;\chi_{1}\;\Phi \left(t+\tau_{1}\right)\;-\;\chi_{2}\;k_{1}\lambda_{8}\left(t+\tau_{2}\right), \end{array}$$

(13) $$\begin{array}{cc} {\dot{\lambda }}_{7}=\; & \lambda_{7}\;\left(\gamma_{D}+\frac{a\;\mu_{D}x_{9}}{{\left(a+x_{7}\right)}^{2}}\right)+\beta_{0}x_{5}\left(\lambda_{5}-\lambda_{6}\right)+\left(\lambda_{9}-\lambda_{10}\right)\frac{a\;\mu_{L}x_{9}}{{\left(a+x_{7}\right)}^{2}}+\frac{c\;\delta \;\lambda_{11}x_{11}}{{\left(c+x_{7}\right)}^{2}}, \end{array}$$

(14) $$\begin{array}{cc} {\dot{\lambda }}_{8}=\; & \gamma_{2}\lambda_{8}+\frac{\eta_{r}\lambda_{4}x_{4}}{{\left(1+x_{8}\right)}^{2}}, \end{array}$$

(15) $$\begin{array}{cc} {\dot{\lambda }}_{9}=\; & A_{4}+\frac{\lambda_{7}\mu_{D}x_{7}}{a+x_{7}}+\lambda_{9}\;\left(\gamma_{L}+G\left(x_{9}\right)+c_{1}x_{11}+\frac{\mu_{L}x_{7}}{a+x_{7}}\right)-\lambda_{10}\;\left(\gamma_{L}+\frac{\mu_{L}x_{7}}{a+x_{7}}\right)\\ \; & +\lambda_{11}\;\left(c_{2}x_{11}-\frac{\rho \;\gamma \;x_{11}}{{\left(\gamma +x_{9}\right)}^{2}}\right)\;-\;\chi_{3}\;2e^{-\gamma_{L}\tau_{3}}\;\lambda_{9}\left(t+\tau_{3}\right)\;G\left(x_{9}\left(t\right)\right), \end{array}$$

(16) $$\begin{array}{cc} {\dot{\lambda }}_{10}=\; & d\;\lambda_{10}, \end{array}$$

(17) $$\begin{array}{cc} {\dot{\lambda }}_{11}=\; & -A_{1}+c_{1}\lambda_{9}x_{9}+\lambda_{11}\;\left(m+c_{2}x_{9}+\frac{\delta x_{7}}{c+x_{7}}-\frac{\rho x_{9}}{\gamma +x_{9}}\right). \end{array}$$

Unless indicated otherwise all state and adjoint variables are evaluated at *t*.

Three structural features dictate the numerical treatment. First, the advanced arguments $\lambda_{8}\left(t+\tau_{2}\right)$, $\lambda_{9}\left(t+\tau_{3}\right)$ and $\Phi (t+\tau_{1})$ are already available when required, since the adjoint system is integrated backward from $t_{f}$; the indicators $\chi_{j}$ switch them off on the terminal window $\left(t_{f}-\tau_{j},\;t_{f}\right]$. Second, only $\lambda_{7}$ enters the control characterization, but $\lambda_{7}$ is coupled to $\lambda_{5},\lambda_{6},\lambda_{9},\lambda_{10},\lambda_{11}$, so the full system must be integrated. Third, ${\dot{\lambda }}_{10}=d\lambda_{10}$ with $\lambda_{10}\left(t_{f}\right)=0$ gives $\lambda_{10}\equiv 0$: the dead-cell compartment neither appears in the objective nor feeds back into the other equations, so it is decoupled from the optimization. We retain it in the implementation as a consistency check on the backward integrator.

### 5.2. Transversality Conditions

Because the objective functional is purely integral, with no terminal cost term $h(x\left(t_{f}\right),t_{f})$, and the final state $x(t_{f})$ is free, the transversality conditions of Pontryagin’s Maximum Principle reduce to the homogeneous form

$$\lambda_{i}\left(t_{f}\right)=0,\;i=1,\ldots ,11.$$

In the absence of a terminal penalty, variations in the final state do not affect the value of the objective functional, so the costates vanish at the terminal time. This reasoning follows the standard treatment in ([27], [Chapter 5, p. 192]). We note for later reference that this is the mechanism by which the optimized infusion is withdrawn as $t\to t_{f}$, a feature discussed in Section 6.

### 5.3. Characterization of the Optimal Control

According to Pontryagin’s Maximum Principle, the optimal control maximizes the Hamiltonian almost everywhere over the admissible set 0≤u(t)≤1. Since

$$\frac{\partial H}{\partial u}=\lambda_{7}\left(t\right)-2A_{5}\;u\left(t\right),\;\frac{\partial^{2}H}{\partial u^{2}}=-2A_{5}<0,$$

the Hamiltonian is strictly concave in *u*, the pointwise maximizer is unique, and the stationary condition yields the unconstrained candidate $u=\lambda_{7}/\left(2A_{5}\right)$. Taking the pointwise bounds into account, the optimal chemotherapy administration rate is obtained by projection onto the admissible interval:

(18) $$u^{*}\left(t\right)=\min\left\{1,\;\max\left\{0,\frac{\lambda_{7}\left(t\right)}{2A_{5}}\right\}\right\}.$$

This expression guarantees feasibility while satisfying the first-order optimality condition. In particular $u^{*}$ is continuous whenever $\lambda_{7}$ is, so the optimal schedule contains no jump discontinuities: the saturated phase $u^{*}\equiv 1$ observed in Section 6 is the interval on which $\lambda_{7}\left(t\right)\geq 2A_{5}$, and the subsequent taper follows the decay of $\lambda_{7}$ toward its zero terminal value.

Two further properties follow, which are not automatic for delayed optimal control problems and which determine the behavior of the numerical scheme.

**Proposition 2** (Regularity and absence of singular arcs)**.** Let $u^{*}$ be optimal with adjoint λ. Then, the following apply:

*(i)*  The maximizer of $u\mapsto H(t,x^{*},x_{\tau }^{*},u,\lambda )$ over [0,1] is unique for every t, and is given by Equation *(18)*.
*(ii)*  $u^{*}$ is Lipschitz continuous on $\left[0,t_{f}\right]$, with $\mathrm{Lip}\left(u^{*}\right)\leq {∥{\dot{\lambda }}_{7}∥}_{\infty }/\left(2A_{5}\right)$.
*(iii)*  *The problem admits no singular arcs.*
*(iv)*  *$\lambda_{10}\equiv 0$, so the dead-cell compartment is decoupled from the optimization.*

**Proof.**  Since $\partial^{2}H/\partial u^{2}=-2A_{5}<0$, the Hamiltonian is strictly concave in *u* and its maximizer over the compact interval [0,1] is unique, which gives (i) and rules out (iii): a singular arc would require ∂H/∂u≡0 on an interval with $\partial^{2}H/\partial u^{2}=0$, which cannot occur for $A_{5}>0$. Assertion (ii) follows because z↦min{1,max{0,z}} is 1-Lipschitz and $\lambda_{7}$ is $C^{1}$ by Equations (7)–(17). Assertion (iv) is immediate from ${\dot{\lambda }}_{10}=d\lambda_{10}$ with $\lambda_{10}\left(t_{f}\right)=0$. □

**Proposition 3** (Uniqueness for a short horizon and convergence of the sweep)**.** *Let K be the compact set of Proposition 1(iii) and let $L_{F}$, $L_{\Lambda }$ denote Lipschitz constants for the right-hand sides of Equation *(1)* and of Equations *(7)–(17)* on K. Define the sweep operator T:U→U by*

$$T\left(u\right)=\min\left\{1,\max\left\{0,\frac{\lambda_{7}\left[u\right]}{2A_{5}}\right\}\right\},$$

*where λ[u] is the adjoint associated with the state trajectory generated by u. Then there exists $C=C(K,L_{F},L_{\Lambda })>0$ such that*

$${∥T\left(u\right)-T\left(v\right)∥}_{\infty }\;\leq \;\frac{C}{2A_{5}}\;t_{f}\;e^{Ct_{f}}\;{∥u-v∥}_{\infty },\;u,v\in U.$$

*Consequently, if $t_{f}$ is small enough that $\kappa :=Ct_{f}e^{Ct_{f}}/\left(2A_{5}\right)<1$, then T is a contraction, the optimal control is unique, and the forward–backward sweep of Section 5.4 converges geometrically to it at rate κ from any admissible initialization. Under the damped update (Equation *(19)*), the corresponding rate is |1−ω+ωκ|, which is less than one for every κ<1 and every ω∈(0,1], and also for κ≥1 provided ω<2/(1+κ).*

**Sketch.**  Standard Gronwall estimates on the forward system give ${∥x\left[u\right]-x\left[v\right]∥}_{\infty }\leq c_{1}t_{f}e^{L_{F}t_{f}}{∥u-v∥}_{\infty }$, the delayed arguments contributing through the method of steps without altering the form of the bound because the histories coincide. A backward Gronwall estimate on Equations (7)–(17), in which the advanced arguments are evaluated on already-determined portions of the solution, gives ${∥\lambda \left[u\right]-\lambda \left[v\right]∥}_{\infty }\leq c_{2}t_{f}e^{L_{\Lambda }t_{f}}{∥x\left[u\right]-x\left[v\right]∥}_{\infty }$. Composing, and using the fact that the projection in Equation (18) is 1-Lipschitz, yields the stated estimate with *C* depending on $c_{1},c_{2},L_{F},L_{\Lambda }$. The contraction mapping theorem then gives uniqueness of the fixed point, which by Proposition 2(i) coincides with the optimal control; the rate for the damped iteration follows from $T_{\omega }=\left(1-\omega \right)\mathrm{Id}+\omega T$. □

**Remark** **4.** *The smallness condition of Proposition 3 is sufficient but far from necessary, and we do not claim it is satisfied for the horizon $t_{f}=10$ used in Section 6; this is the same limitation identified for the undelayed case in [17]. What the proposition provides is a single estimate governing both the well-posedness of the optimization and the behavior of the numerical scheme, together with the explicit role of the exposure weight $A_{5}$: increasing $A_{5}$ contracts the sweep. The convergence observed at the parameter values used is reported in Section 6.3 and is consistent with κ<1.*

### 5.4. Numerical Computation of the Optimal Control

Because the control depends on both the state and adjoint variables, the optimality system is solved numerically by a forward–backward sweep iterative procedure. The delayed setting requires two modifications of the classical scheme: the forward solve must carry a history buffer, and the backward solve must access adjoint values at the advanced arguments $t+\tau_{j}$. The latter is the reason a fixed-step scheme on a stored grid is used rather than an adaptive solver, which cannot supply values at times it has not yet reached.

#### 5.4.1. Discretization

The horizon $\left[0,t_{f}\right]$ is discretized with a uniform step *h*; delayed and advanced values are obtained from the stored trajectory by interpolation, the history being constant and equal to φ for t≤0. Both sweeps use a classical fourth-order Runge–Kutta scheme. The baseline step is $h=10^{-3}$ days, giving $N=10^{4}$ steps; grid independence is documented in Section 6.

#### 5.4.2. Algorithm

1. **Initialization.** Set k=0 and choose $u^{\left(0\right)}\equiv 0.1$ on $\left[0,t_{f}\right]$:
2. **Forward sweep.** Using $u^{\left(k\right)}$, integrate state System (1) forward from t=0 to $t=t_{f}$ with the prescribed history, storing $x^{\left(k\right)}$ at every node.
3. **Backward sweep.** Using $x^{\left(k\right)}$, integrate adjoint Systems (7)–(17) backward from $t=t_{f}$ subject to
  $$\lambda_{i}\left(t_{f}\right)=0,\;i=1,\ldots ,11.$$
  On the terminal window $\left(t_{f}-\tau_{j},t_{f}\right]$ the corresponding advanced term is set to zero through $\chi_{j}$; elsewhere $\lambda_{8}\left(t+\tau_{2}\right)$, $\lambda_{9}\left(t+\tau_{3}\right)$ and $\Phi (t+\tau_{1})$ are read from nodes already computed in the current backward pass.
4. **Control update with damping.** Form the pointwise maximizer
  $${\tilde{u}}^{\left(k\right)}\left(t\right)=\min\;\left\{1,\max\;\left\{0,\frac{\lambda_{7}^{\left(k\right)}\left(t\right)}{2A_{5}}\right\}\right\}$$
  and update by convex combination
  (19) $$u^{\left(k+1\right)}=\omega \;{\tilde{u}}^{\left(k\right)}+\left(1-\omega \right)\;u^{\left(k\right)},\;\omega \in \left(0,1\right].$$
  The undamped scheme corresponds to ω=1; we use ω=0.5 throughout.
5. **Stopping criterion.** Terminate when the relative sup-norm increment satisfies
  (20) $$\frac{∥u^{\left(k+1\right)}-u^{\left(k\right)}{∥}_{\infty }}{\max\{∥u^{\left(k+1\right)}{∥}_{\infty },\;\epsilon_{0}\}}<\varepsilon ,$$
  with tolerance $\varepsilon =10^{-6}$ and $\epsilon_{0}=10^{-12}$ guarding the degenerate case u≡0.

The final iterate is taken as an approximation of the optimal control and is used in the simulations presented in Section 6.

#### 5.4.3. Convergence

Convergence of the forward–backward sweep is not automatic: McAsey, Mou and Han [17] showed that the iteration is a contraction provided the right-hand sides and the control characterization are Lipschitz and the horizon is sufficiently short relative to the Lipschitz constants, and that divergence can occur otherwise. Proposition 3 gives the corresponding estimate for the present delayed problem, and Section 6 reports the observed convergence history together with a grid-refinement study.

#### 5.4.4. A Numerical Detail

The immune death term $\delta x_{11}x_{7}/\left(c+x_{7}\right)$ has half-saturation $c=7.14\times 10^{-7}$, so its derivative with respect to $x_{7}$ is of order $\delta /c\approx 2\times 10^{3}$ at $x_{7}=0$ and negligible thereafter. Since $x_{7}\left(0\right)=0$ exactly, the adjoint right-hand side carries a removable spike at the initial node, which a fixed-step scheme cannot resolve; we therefore set $u^{\left(k+1\right)}\left(0\right)=u^{\left(k+1\right)}\left(h\right)$ at each iteration. The effect is confined to a single node and does not affect the reported results.

## 6. Numerical Results

### 6.1. Numerical Implementation

The simulations were performed in MATLAB R2016a using the fixed-step scheme described in Section 5.4. The state and adjoint systems were integrated with a classical fourth-order Runge–Kutta method on a uniform grid of step $h=10^{-3}$ days, giving $N=10^{4}$ steps on the horizon $\left[0,t_{f}\right]$ with $t_{f}=10$ days; delayed and advanced arguments were obtained from the stored trajectory, the initial history being taken constant on $\left[-\tau_{\max},0\right]$. The forward–backward sweep used the damped update (Equation (19)) with ω=0.5 and terminated at the relative tolerance $\varepsilon =10^{-6}$ after 23 iterations. Model parameters are those of Table 8; the initial history was

φ≡(1.0,0.1,0.5,0.1,0.3,0.2,0,0.1,1.5,0,0.5),

representing a biologically plausible immune and leukemic state in the absence of treatment.

#### Comparison Scenarios

A comparison against an untreated baseline and a single low constant dose does not by itself establish that the *shape* of the optimal schedule is responsible for any observed improvement, since the optimal schedule delivers a larger cumulative dose than a low constant infusion. Writing

$$E\left(u\right):=\int_{0}^{t_{f}}u\left(t\right)\;dt$$

for the cumulative normalized drug exposure, the computed optimal control satisfies $E(u^{*})=4.4596$, so that a constant infusion delivering the same total drug is

$$\bar{u}=\frac{E(u^{*})}{t_{f}}=0.4460.$$

This is more than four times the constant dose u=0.1 considered previously. We therefore compare four strategies:

- **No treatment:** u(t)=0, E=0;
- **Low constant dose:** u(t)=0.1, E=1.0;
- **Exposure-matched constant dose:** $u\left(t\right)=\bar{u}=0.4460$, E=4.4596;
- **Optimal control:** $u^{*}\left(t\right)$, E=4.4596.

The third scenario isolates the contribution of dose *timing*: any difference between it and the optimal schedule cannot be attributed to a difference in total drug administered.

**Figure 1:**
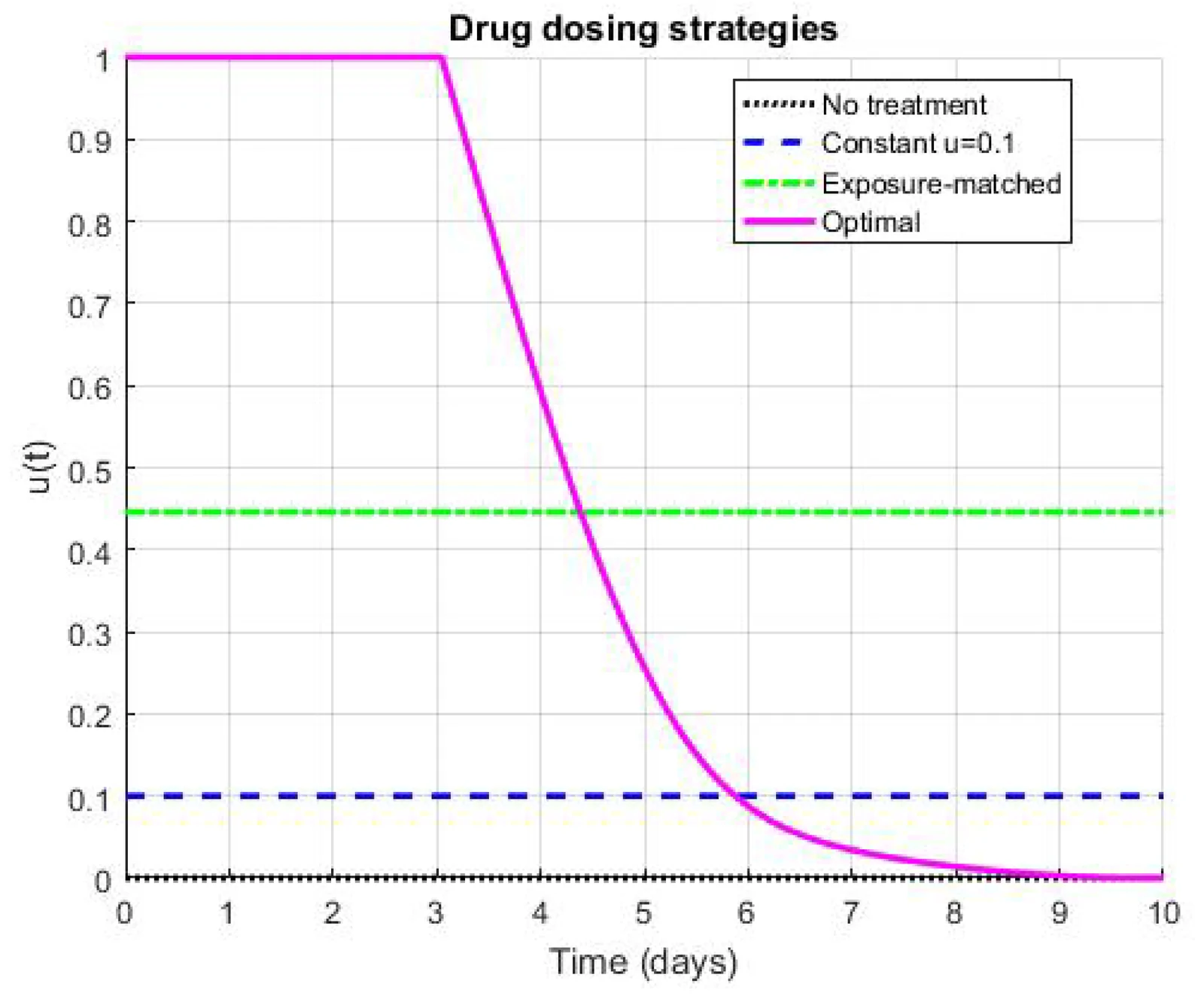
Comparison of drug dosing strategies. The optimal control and the exposure-matched constant dose deliver identical cumulative exposure E=4.4596.

Figure 1 shows the four dosing profiles. The optimal control applies the maximal admissible dose $u^{*}=1$ during the initial phase, up to approximately day 3, and then decreases smoothly toward zero, becoming negligible after about day 8. This bang-then-taper structure reflects the optimality condition (Equation (18)): the control saturates on the interval where $\lambda_{7}\left(t\right)\geq 2A_{5}$, and the subsequent taper follows the decay of $\lambda_{7}$ toward its zero terminal value. The exposure-matched protocol distributes the same total drug uniformly at $\bar{u}=0.4460$.

**Figure 2:**
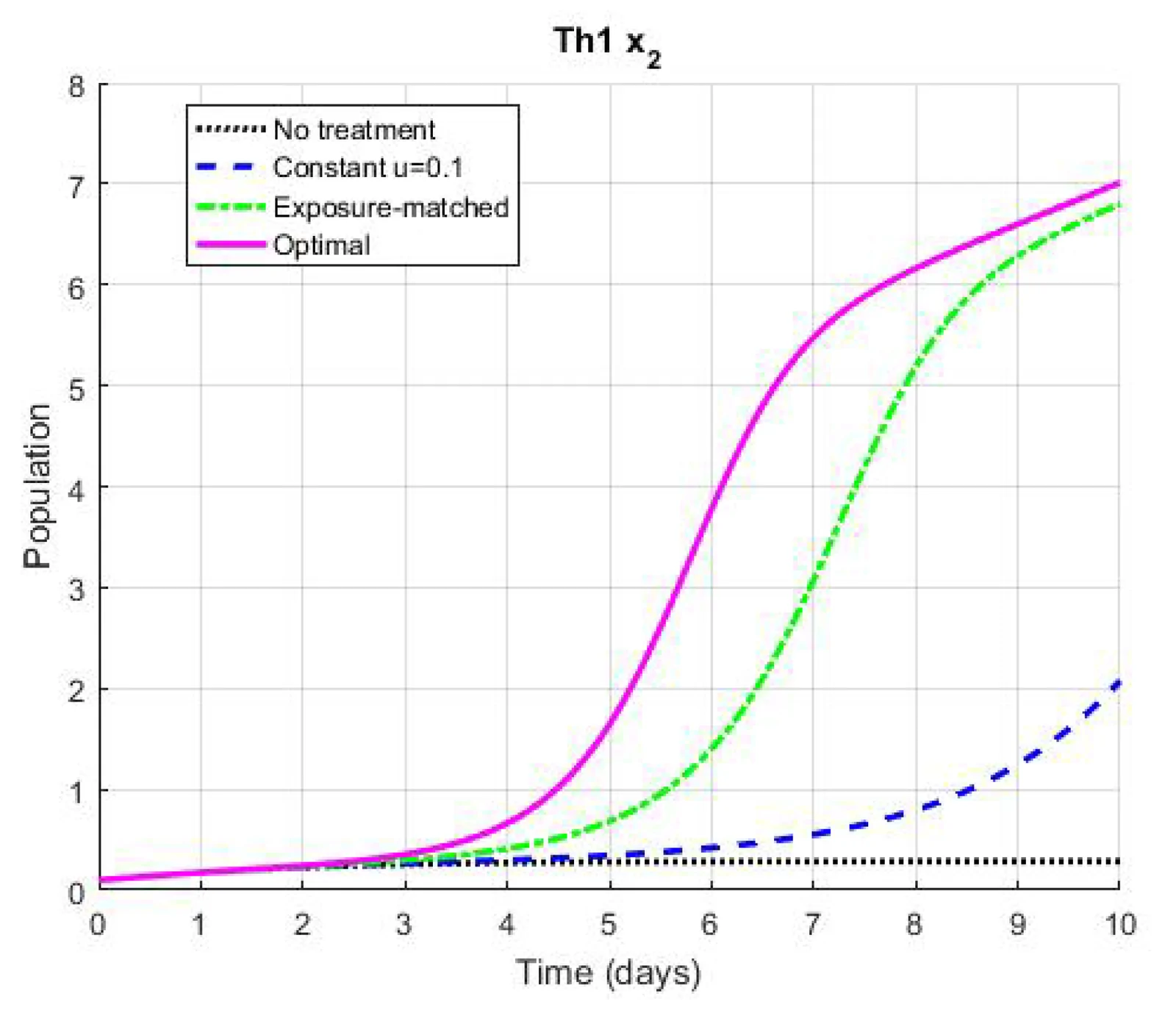
Effector T cells $x_{2}\left(t\right)$ (Th1) under the four dosing strategies.

Figure 2 shows the Th1 effector population. Under optimal control the Th1 cells remain low during the first days and then expand rapidly, reaching 7.011 at the end of the horizon; the exposure-matched constant dose produces a similar terminal level of 6.803 but with the expansion displaced later in time. The low constant dose produces only a modest late increase to 2.073, and the untreated case remains essentially flat at 0.286. The distinguishing feature of the optimal schedule is therefore not the terminal Th1 level, which the exposure-matched protocol nearly attains, but the timing of the expansion.

**Figure 3:**
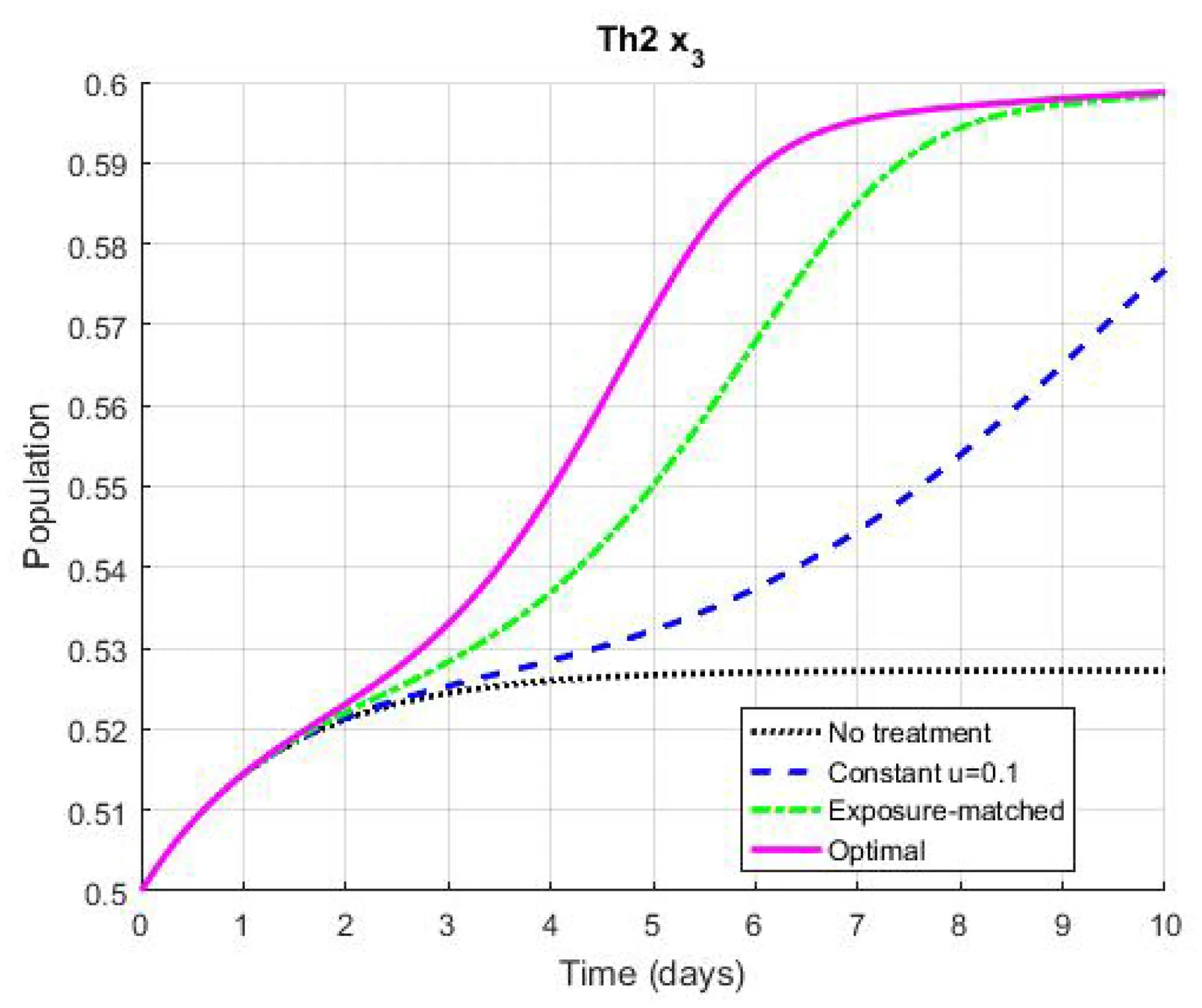
Suppressor T cells $x_{3}\left(t\right)$ (Th2) under the four dosing strategies.

Figure 3 shows the Th2 population. In absolute terms the Th2 level rises modestly under all four scenarios, from 0.5 to between 0.527 and 0.599, and is in fact slightly highest under optimal control. The therapeutic benefit therefore lies not in suppressing Th2 in absolute terms but in the *relative* balance between Th1 and Th2. The terminal ratio $x_{2}/x_{3}$ is 0.54 without treatment, 3.60 under the low constant dose, 11.37 under the exposure-matched dose and 11.71 under optimal control. More informative than the terminal ratio is the time at which Th1 overtakes Th2: this occurs at day 3.74 under optimal control against day 4.61 under the exposure-matched protocol and day 6.96 under the low constant dose, and never within the horizon in the untreated case.

**Figure 4:**
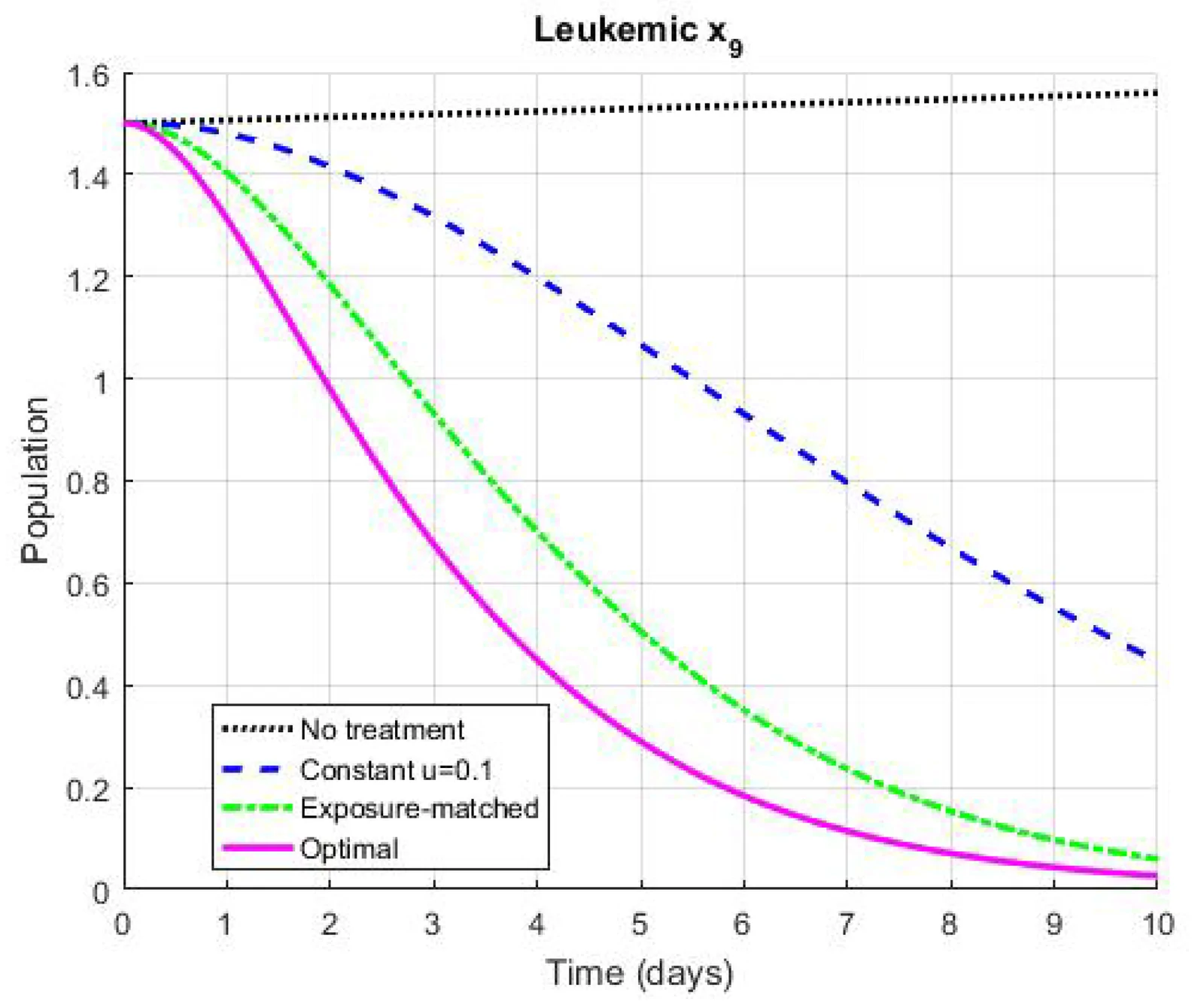
Leukemic burden $x_{9}\left(t\right)$ under the four dosing strategies.

Figure 4 shows the leukemic burden. Under optimal control the population decreases from its initial value of 1.5 to 0.0267, a reduction of 98.3% relative to the untreated terminal value of 1.56; the exposure-matched constant dose reaches 0.0601, a reduction of 96.1%, while the low constant dose reaches only 0.4474 and the untreated case shows slight continued progression. Thus, at equal cumulative exposure, the optimal schedule attains a terminal leukemic burden lower by a factor of 2.25, but the larger part of the improvement over the low constant dose is attributable to total exposure rather than to scheduling.

The separation between the two exposure-matched strategies is more pronounced in the rate of response than in the endpoint. Writing $t_{50}$ and $t_{90}$ for the times at which the leukemic burden falls below 0.75 and 0.15, corresponding to reductions of 50% and 90% from its initial value, the optimal schedule attains $t_{50}=2.74$ days and $t_{90}=6.43$ days, against $t_{50}=3.78$ and $t_{90}=8.05$ days for the exposure-matched constant dose—reductions of 28% and 20%, respectively. The low constant dose reaches $t_{50}=7.36$ days and does not achieve a 90% reduction within the horizon.

**Figure 5:**
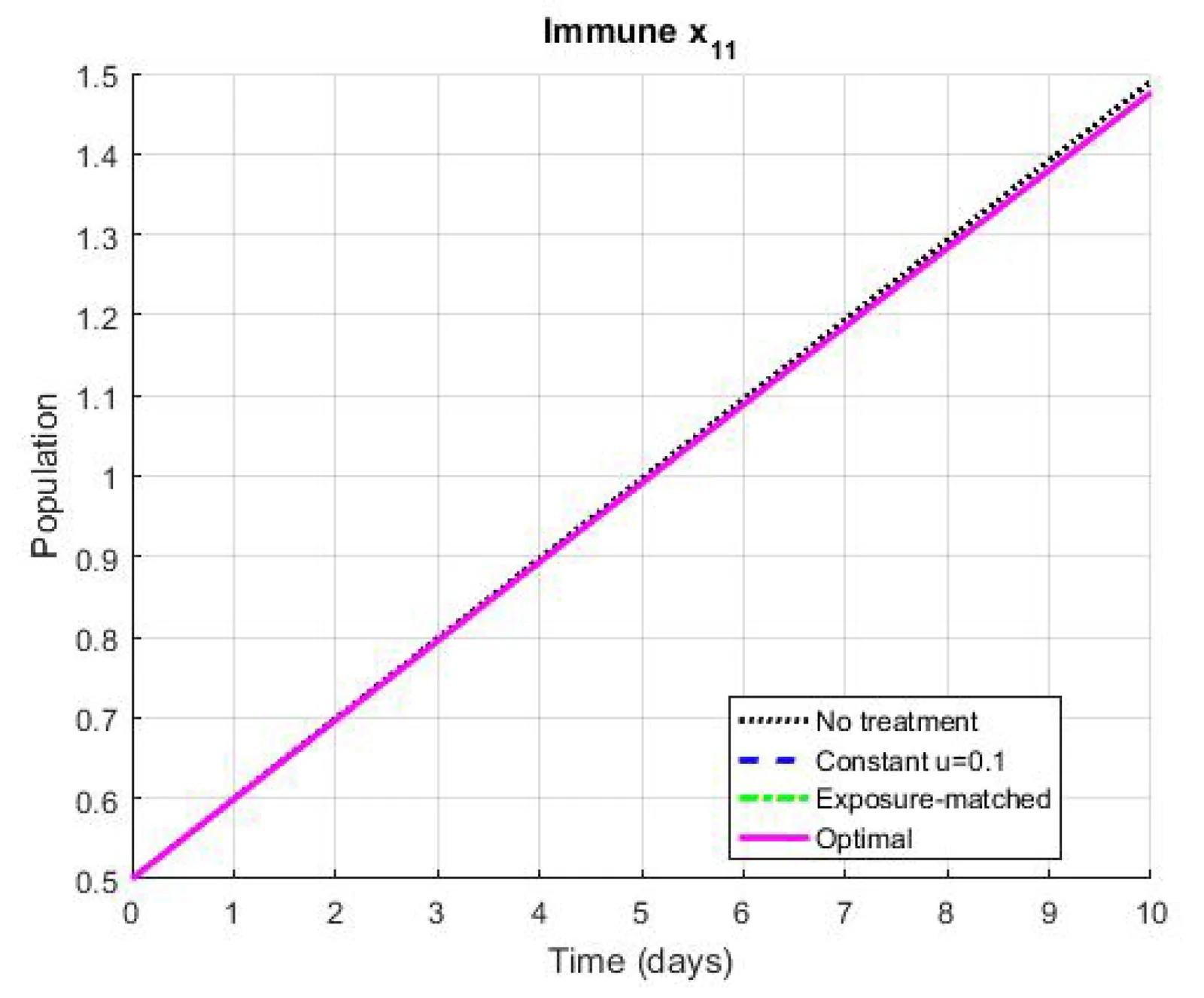
Immune effector population $x_{11}\left(t\right)$ under the four dosing strategies. The four trajectories are nearly coincident and therefore overlap; this near-identity across dosing strategies is the point of the figure and is discussed in the text.

Figure 5 shows the immune effector population. The four scenarios produce nearly indistinguishable trajectories, the population growing steadily from 0.5 to approximately 1.48 in all cases, with the untreated case marginally highest. As discussed in the limitations, this reflects the fact that in the present parameter regime the interaction coefficients $c_{1}$, $c_{2}$ and ρ are several orders of magnitude smaller than the supply and decay rates *s* and *m*, so that the effector equation is dominated by its intrinsic supply–decay balance. The practical implication is favorable but must be stated precisely: the optimal strategy achieves substantial leukemic reduction without measurable loss of immune effector competence, and the reduction is attributable to the direct cytotoxic action of the drug rather than to enhanced immune-mediated clearance.

Table 1 summarizes the comparison, and read together with the exposure-matched arm it supports a more precise statement of the contribution than a comparison against an untreated baseline alone would allow.

The objective value provides the primary ordering: *J* increases from −8.03 under no treatment to −0.65 under the low constant dose, 17.18 under the exposure-matched constant dose, and 24.15 under optimal control. The last two are the informative pair, since they are attained at identical cumulative exposure: the optimal schedule improves the objective by 40.5% over a constant infusion delivering the same total drug. This ordering is structural rather than empirical—$u^{*}$ maximizes Equation (3) by construction, so no admissible control can exceed it—and it therefore serves as a consistency check on the computed solution as much as a comparison between protocols. What the exposure-matched arm establishes is the *magnitude* of the gap: redistributing a fixed quantity of drug over time is worth some forty percent in the quantity the problem was posed to maximize.

The population-level measures show where that gap originates. At equal cumulative exposure the optimal schedule attains a lower terminal leukemic burden (0.0267 against 0.0601, a factor of 2.25), a marginally higher terminal Th1/Th2 ratio, and a lower minimum combined divergence; but the differences in terminal quantities are modest, and the exposure-matched protocol reaches a broadly comparable final configuration. The consistent separation is in timing. Th1 dominance is established 0.87 days sooner, a 50% leukemic reduction 1.04 days sooner, a 90% reduction 1.62 days sooner, and the minimum of the combined divergence 1.37 days sooner. The benefit of adaptive scheduling in this model is therefore principally one of *rate of response at fixed drug exposure* rather than of eventual position.

One quantity runs the other way and should be recorded explicitly. The terminal combined divergence is higher under optimal control (0.3327) than under the exposure-matched constant dose (0.2924). This is a consequence of the free terminal state: as $\lambda_{7}$ approaches its zero terminal value, the optimized infusion is withdrawn, and the leukemic population begins to recover after approximately day 6, whereas the constant infusion continues to act until $t_{f}$. The minimum of the divergence, attained at day 5.82 under optimal control against day 7.19 under the exposure-matched dose, is both lower and earlier, and Section 6.5 shows that both orderings—optimal lower at the minimum, higher at $t_{f}$—persist across twenty alternative reference profiles. The effect is thus a property of the formulation rather than of the diagnostic: a terminal cost on the leukemic population would remove it, and we regard this as a natural extension rather than a defect of the present formulation.

### 6.2. Sensitivity Analysis

Because the model parameters are drawn from heterogeneous sources and several were calibrated rather than measured (Section 8), the conclusions must be shown to be robust to plausible variation. We performed a one-at-a-time study in four groups: kinetic parameters, delays, initial leukemic load, and objective weights. For each perturbed configuration the full optimality system was re-solved to the tolerance of Section 5.4, and five outcomes were recorded: the terminal leukemic burden $x_{9}\left(t_{f}\right)$, the cumulative exposure $E(u^{*})$, the minimum combined divergence $D_{\mathrm{tot}}^{\min}$, and the times $t_{50}$, $t_{90}$ at which the leukemic burden falls below 0.75 and 0.15, respectively. For the first three, we report the normalized sensitivity index

$$S_{\pi }^{\;Q}\;\approx \;\frac{Q\left(\pi (1+\epsilon )\right)-Q\left(\pi (1-\epsilon )\right)}{2\epsilon \;Q(\pi )},\;\epsilon =0.2,$$

so that |S|>1 indicates amplification of the relative perturbation; for $t_{50}$ and $t_{90}$ we report the absolute shift in days, which is more directly interpretable.

#### 6.2.1. Kinetic Parameters

Table 2 reports the sixteen kinetic parameters examined. The results are strongly concentrated: a single parameter dominates every outcome.

Three features of Table 2 deserve comment.

First, the drug-induced leukemic death rate $\mu_{L}$ is the only parameter whose perturbation is amplified: $S_{\mu_{L}}^{x_{9}\left(t_{f}\right)}=-4.53$, so a 20% uncertainty in $\mu_{L}$ produces roughly a 90% change in terminal leukemic burden, and shifts $t_{90}$ by about a day. This is expected, since $\mu_{L}$ governs the direct cytotoxic term through which the control acts, and it identifies $\mu_{L}$ as the parameter most in need of experimental determination. Notably, the effect on $E(u^{*})$ is two orders of magnitude smaller (−0.024): the *schedule* is largely insensitive to $\mu_{L}$ even though the *outcome* is not.

Second, all remaining parameters have |S|<0.32, and most are below 0.01. In particular the parameters governing the immune–leukemic interaction, $c_{1}$, $c_{2}$, ρ, *m* and δ, have sensitivity indices indistinguishable from zero across all five outcomes. This provides quantitative confirmation of the structural observation recorded in the limitations: in the present parameter regime the immune effector compartment is dynamically decoupled from the leukemic dynamics, and the reported therapeutic effect is attributable entirely to the direct cytotoxic action of the drug.

Third, θ has $S\approx 10^{-4}$ across all outcomes. Since θ=100 while the leukemic burden never exceeds 1.6, the Hill factor ${\left(1+{\left(x_{9}/\theta \right)}^{2}\right)}^{-1}$ appearing in Equation (2) remains within $3\times 10^{-4}$ of unity throughout the simulation, so the saturating nonlinearity in the self-renewal flux is inactive in this regime and $\Psi (x_{9})$ reduces to $\beta_{L}x_{9}^{2}$ to four significant figures. The leukemic equation therefore operates in its effectively quadratic range, and the results reported here do not probe the density-dependence that Ψ was introduced to represent; this should be borne in mind when interpreting the model.

A separate observation concerns the allergy axis. The Treg inhibition rate $\eta_{r}$ has a negligible effect on the leukemic outcomes but the largest effect of any kinetic parameter on the minimum combined divergence, $S_{\eta_{r}}^{D^{\min}}=0.212$, with κ and ϕ next. These are precisely the parameters governing the Th1/Th2/Treg balance, so the two therapeutic axes are sensitive to disjoint sets of parameters—a structural separation that supports treating them as distinct objectives.

#### 6.2.2. Delays

Table 3 reports the sensitivity of the five outcomes to the three delays. The leukemic cell-cycle delay $\tau_{3}$ is the only influential delay, with $S_{\tau_{3}}^{x_{9}\left(t_{f}\right)}=0.45$; this is unsurprising, since $\tau_{3}$ enters the leukemic equation both through the shifted argument and through the amplification factor $2e^{-\gamma_{L}\tau_{3}}$. The transport and cytokine delays $\tau_{1}$ and $\tau_{2}$ have indices below 0.01 on every outcome.

#### 6.2.3. Initial Leukemic Load

Table 4 reports the dependence of the computed schedule on the initial leukemic burden. Across a sixfold variation in initial disease burden, the bang-then-taper structure of the optimal control is preserved, and the switching time varies only between 2.97 and 3.18 days—a range of 7% for a 500% change in initial condition. The cumulative exposure likewise varies by less than 3%. The optimal *schedule* is therefore remarkably insensitive to the initial leukemic load, while the terminal burden scales approximately linearly with it. The minimum combined divergence is non-monotonic, attaining its lowest value at the baseline load $x_{9}\left(0\right)=1.5$; this reflects the two axes trading off, since a smaller initial burden leaves the allergy axis relatively further from its target.

#### 6.2.4. Objective Weights

Table 5 reports the effect of the four objective weights. The weights separate sharply into two groups. The exposure weight $A_{5}$ and the Th1 reward $A_{2}$ substantially reshape the schedule: increasing $A_{5}$ from 0.5 to 5 reduces cumulative exposure from 5.05 to 2.79 and shortens the saturated phase from four days to nothing, the control ceasing to saturate at all at $A_{5}=5$; increasing $A_{2}$ over the same range has the opposite effect, raising exposure from 3.84 to 5.68. The behavior of $A_{5}$ is the one predicted directly by optimality Condition (18), in which $A_{5}$ rescales the threshold $\lambda_{7}\geq 2A_{5}$ at which the control saturates, and its appearance in the numerics is a useful consistency check on the optimality system.

By contrast, the leukemic penalty $A_{4}$ and the Th2 penalty $A_{3}$ have little effect. A tenfold increase in $A_{4}$ changes the terminal leukemic burden by only 4%, and a tenfold increase in $A_{3}$ changes it in the fourth decimal place; $A_{3}$ is essentially inert because, as Figure 3 shows, the Th2 population varies by less than 15% across all strategies, so the term $-A_{3}x_{3}$ contributes almost no gradient to the optimization. This is worth stating plainly: the Th2 penalty, though motivated by the allergy objective that partly defines the problem, does not materially influence the computed schedule in this parameter regime.

Finally, $t_{50}$ is invariant at 2.734 days across every weight configuration except $A_{5}=5$. The rate of early response is therefore a property of the dynamics and the saturation constraint rather than of the particular trade-off encoded in the weights, which reinforces the interpretation of the timing advantage reported in Table 1 as a robust feature of the optimal schedule.

#### 6.2.5. Summary

Across all perturbations considered, the qualitative structure of the optimal schedule—an initial saturated phase of approximately three days followed by a smooth taper—and the ordering of the four dosing strategies with respect to every outcome measure were preserved, the single exception being $A_{5}=5$, at which the exposure penalty is high enough that saturation never occurs. Quantitatively, only $\mu_{L}$ amplifies its relative perturbation, and only $\mu_{L}$, $\beta_{L}$, $\gamma_{L}$ and $\tau_{3}$ have indices above 0.1 on any outcome. The calibrated parameters $\beta_{L}$, $\gamma_{L}$ and θ discussed in Section 8 have indices of 0.32, −0.11 and $10^{-4}$, respectively, so the conclusions are not sensitive to the calibration choices at the level of a ±20% uncertainty.

### 6.3. Convergence of the Numerical Scheme

**Figure 6:**
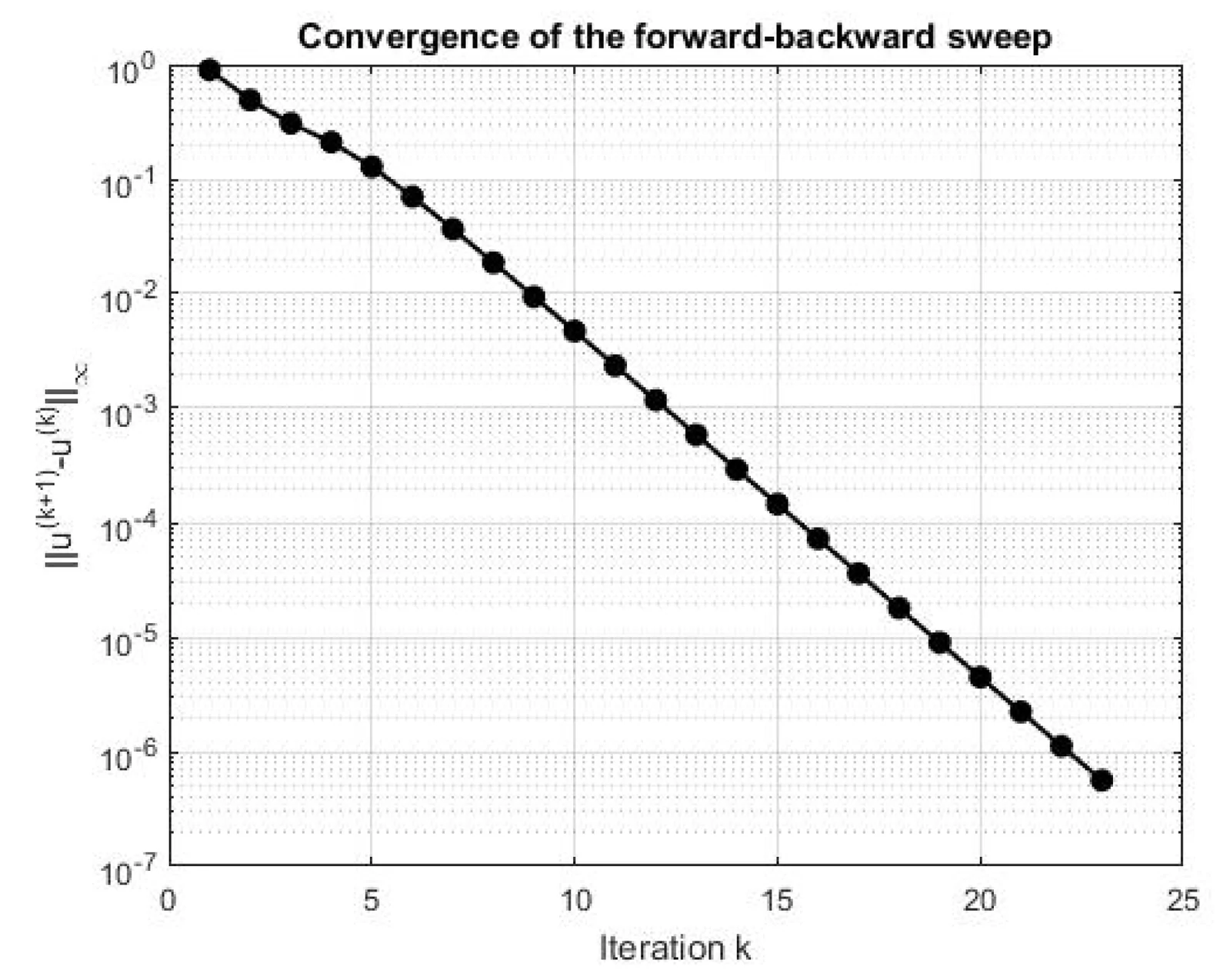
Convergence of the forward–backward sweep: sup-norm of the control increment $∥u^{\left(k+1\right)}-u^{\left(k\right)}{∥}_{\infty }$ against iteration index *k*, logarithmic vertical axis, at the baseline parameter set with damping ω=0.5.

Figure 6 reports the convergence history. The control increment decreases from $9.0\times 10^{-1}$ at the first iteration to $5.6\times 10^{-7}$ at the twenty-third, at which point the relative tolerance $\varepsilon =10^{-6}$ was met. The decay is linear on the logarithmic axis over six orders of magnitude, with an observed contraction factor of approximately 0.55 per iteration. This is consistent with the estimate of Proposition 3: for the damped iteration the predicted rate is |1−ω+ωκ|, so that the observed value corresponds to κ≈0.1 at the parameter values used, comfortably inside the contraction regime. We emphasize that this is empirical verification at one parameter set and not a proof that the sufficient condition of Proposition 3 holds at $t_{f}=10$.

Table 6 reports the effect of successive grid refinement. The terminal leukemic burden and the cumulative exposure agree to six significant figures across a fourfold change in step size, and the sup-norm difference between the controls computed on successive grids falls from $1.43\times 10^{-4}$ to $2.71\times 10^{-7}$; the corresponding $L^{2}$ differences are $9.66\times 10^{-6}$ and $2.92\times 10^{-7}$, a reduction consistent with at least fourth-order behavior. The larger difference at the coarsest refinement is localized at the instant the control leaves saturation, where $u^{*}$ has a corner whose resolution improves rapidly with the mesh; away from it the agreement is uniform. All quantities reported in this paper are therefore insensitive to the discretization at the working step $h=10^{-3}$.

A further observation concerns the iteration count, which is 23 on all three grids. The number of sweeps required to meet Tolerance (20) is thus independent of the discretization, as the estimate of Proposition 3 predicts: the contraction factor is a property of the continuous optimality system and of the damping parameter, not of the mesh on which it is approximated.

### 6.4. Information-Theoretic Characterization of the Therapeutic Goal

The therapeutic goal admits a natural information-theoretic description, which we develop in this section. Before introducing the relevant quantities, we make their status precise since it bears directly on what the control problem is taken to optimize. Objective Functional (3) aggregates four populations measured in different units, and its numerical value is therefore interpretable only relative to a stated normalization (Remark 2). A divergence between normalized distributions, by contrast, is dimensionless, invariant under rescaling of the underlying populations, and directly comparable across strategies and across the two therapeutic axes. In the present section and in Section 6.5 and Section 6.6 these quantities are evaluated *along* the trajectories generated by each dosing strategy and serve as diagnostics; the control being diagnosed is the one determined by Equation (3), in which they play no part. Section 6.7 then treats the complementary problem in which the combined divergence is itself the running cost, and compares the two resulting schedules.

The treatment studied here pursues two competing objectives that can each be expressed as a balance between cell populations. The first is the control of treatment-induced allergic reactions, governed by the adaptive T-helper compartment: a Th1-dominant configuration is associated with low hypersensitivity risk, whereas a shift toward Th2 dominance promotes allergic inflammation, with regulatory T cells (Treg) modulating this balance. The second is the control of the disease itself, governed by the relative abundance of immune effector cells and leukemic cells: an immune-dominant configuration corresponds to effective tumor suppression. Both balances admit a natural information-theoretic formulation, which we develop on two distinct axes.

#### 6.4.1. Allergy Axis

Restricting attention to the adaptive T-helper compartment $\left\{x_{2},x_{3},x_{4}\right\}$ (Th1, Th2, Treg), we define the normalized subpopulation distribution

$$p_{i}^{A}\left(t\right)=\frac{x_{i}\left(t\right)}{\sum_{j\in J_{A}}x_{j}\left(t\right)},\;J_{A}=\left\{2,3,4\right\},\;i\in J_{A},$$

with Shannon entropy

$$S_{A}\left(t\right)=-\sum_{i\in J_{A}}p_{i}^{A}\left(t\right)\;\ln p_{i}^{A}\left(t\right),\;0\leq S_{A}\left(t\right)\leq \ln3,$$

which quantifies the diversity and regulatory order of the helper T-cell response. To measure proximity to an allergy-protective profile, we introduce a reference distribution $q_{A}=\left(0.70,\;0.10,\;0.20\right)$ and evaluate the relative entropy

$$D_{A}\left(t\right)=D_{\mathrm{KL}}\;\left(p^{A}\left(t\right)\;∥\;q_{A}\right)=\sum_{i\in J_{A}}p_{i}^{A}\left(t\right)\;\ln\frac{p_{i}^{A}\left(t\right)}{q_{A,i}}.$$

The three components of $q_{A}$ encode Th1 predominance, since a Th1-skewed helper compartment is associated with low hypersensitivity risk [9,10]; a small but nonzero Th2 fraction, since complete Th2 depletion is neither physiological nor a therapeutic goal; and an intermediate regulatory fraction, since Treg cells must remain sufficient to restrain the response without suppressing anti-tumor immunity. These are qualitative immunological targets rather than calibrated values, and Section 6.5 verifies that the conclusions do not depend sensitively on them.

#### 6.4.2. Leukemia Axis

Analogously, restricting attention to the disease-control pair $\left\{x_{11},x_{9}\right\}$ (immune effector and leukemic cells), we define

$$p_{i}^{L}\left(t\right)=\frac{x_{i}\left(t\right)}{x_{11}\left(t\right)+x_{9}\left(t\right)},\;i\in J_{L}=\left\{11,9\right\},$$

and evaluate the relative entropy to an immune-dominant reference $q_{L}=\left(0.85,\;0.15\right)$, in the order (immune, leukemic),

$$D_{L}\left(t\right)=D_{\mathrm{KL}}\;\left(p^{L}\left(t\right)\;∥\;q_{L}\right)=\sum_{i\in J_{L}}p_{i}^{L}\left(t\right)\;\ln\frac{p_{i}^{L}\left(t\right)}{q_{L,i}}.$$

This reference encodes immune predominance over a residual leukemic fraction rather than complete eradication, which is not attainable within the horizon considered.

#### 6.4.3. Combined Therapeutic Divergence

Each divergence is nonnegative and vanishes only when the corresponding balance coincides with its healthy target. To summarize both goals in a single quantity we define the combined therapeutic divergence

$$D_{\mathrm{tot}}\left(t\right)=D_{A}\left(t\right)+D_{L}\left(t\right),$$

which measures the total information-theoretic distance of the immune–leukemic state from a simultaneously allergy-protective and disease-controlling configuration.

The optimal control $u^{*}\left(t\right)$ derived in Section 5.4 does not act on $D_{\mathrm{tot}}$ directly, since the divergences do not appear in Equation (3); what follows is therefore an assessment of how far the schedule obtained from the population-based objective happens to advance the information-theoretic one. The entropy $S_{A}\left(t\right)$ is examined separately in Section 6.6, where it is shown to carry information that the divergence alone does not.

**Figure 7:**
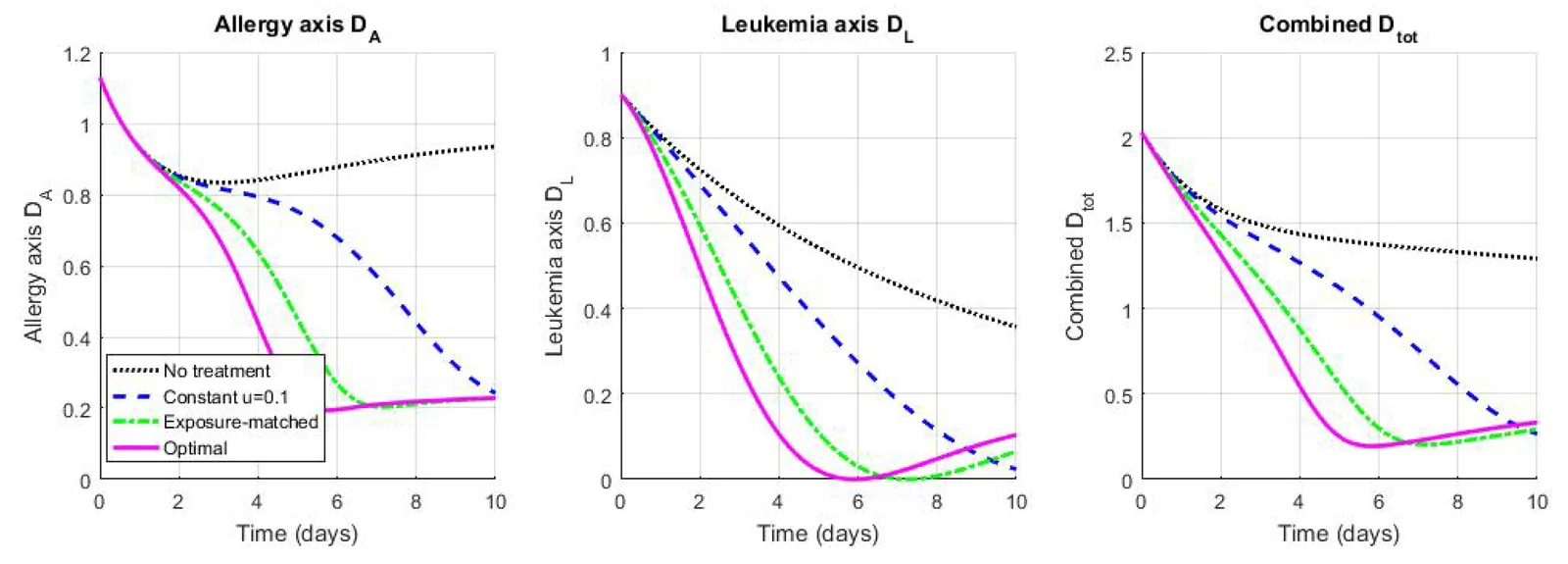
Information-theoretic diagnostics of the therapeutic goal for the four dosing strategies. **Left**: relative entropy $D_{A}\left(t\right)$ on the allergy axis (Th1/Th2/Treg distribution to a Th1-favorable reference $q_{A}$). **Center**: relative entropy $D_{L}\left(t\right)$ on the leukemia axis (immune/leukemic distribution to an immune-dominant reference $q_{L}$). **Right**: combined therapeutic divergence $D_{\mathrm{tot}}\left(t\right)=D_{A}\left(t\right)+D_{L}\left(t\right)$.

Figure 7 reports the two axis divergences and their sum for the four dosing strategies. On the allergy axis (left), all cases begin from a large divergence, $D_{A}\approx 1.13$, reflecting an initially mixed helper T-cell configuration with elevated allergic potential. The optimal control produces the fastest decrease, falling to approximately 0.23 by day 5 and remaining close to that level thereafter; the exposure-matched constant dose follows the same descent displaced by roughly a day, the low constant dose declines gradually and reaches a comparable value only at the end of the horizon, and the untreated trajectory initially decreases but subsequently drifts *away* from the target, rising to 0.93 as the disease persists. It is worth noting that $D_{A}$ does not approach zero under any strategy: as discussed in Section 6.6, the treated trajectories overshoot the Th1 fraction prescribed by $q_{A}$ rather than converging to it.

On the leukemia axis (center), all cases start at $D_{L}\approx 0.91$. The optimal control drives the immune/leukemic balance to the target fastest, with $D_{L}$ reaching a minimum near zero at approximately day 6.3; thereafter, the divergence increases again, to about 0.10 at $t_{f}$, as the tapering infusion permits partial leukemic recovery. The exposure-matched constant dose reaches its minimum about a day and a half later but, because it continues to deliver drug until $t_{f}$, remains close to the target at the terminal time. The low constant dose declines steadily throughout, and the untreated case remains far from the target, consistent with continued tumor progression.

The combined divergence (right) inherits both features, and the comparison between the two exposure-matched strategies depends on which instant is examined. The optimal schedule attains the lower *minimum*, $D_{\mathrm{tot}}^{\min}=0.194$ at day 5.82, against 0.203 at day 7.19 for the exposure-matched constant dose: lower, and reached 1.37 days earlier. At the *terminal* time, the ordering reverses, $D_{\mathrm{tot}}\left(t_{f}\right)=0.333$ under optimal control against 0.292 under the exposure-matched dose, for the reason given above. Both orderings are stable under variation of the reference profiles, as documented in Section 6.5. The untreated case remains highest throughout by a wide margin, ending at 1.292.

These trajectories admit a direct biological interpretation, and one qualification. The optimal control, by adjusting the infusion rate in response to the evolving state, steers the helper T-cell distribution toward a Th1-favorable configuration and the immune/leukemic balance toward immune dominance more rapidly than any of the alternatives, and it does so at cumulative drug exposure identical to that of the exposure-matched protocol. The qualification is that this advantage is one of rate rather than of eventual position: given the same total drug, a constant infusion reaches a comparable configuration roughly a day and a half later, and—because it is still acting at $t_{f}$—holds it slightly better at the end of a fixed horizon. The diagnostics therefore support the conclusion that adaptive dosing advances both therapeutic objectives faster, but not the stronger conclusion that it advances them further within this model and horizon.

### 6.5. Robustness with Respect to the Reference Profiles

Since $q_{A}$ and $q_{L}$ are prescribed rather than estimated, the comparison between dosing strategies must be shown not to depend on the particular choice. We recomputed $D_{A}$, $D_{L}$ and $D_{\mathrm{tot}}$ along all four trajectories for every combination of

$$q_{A}\in \left\{(0.70,0.10,0.20),\;(0.60,0.20,0.20),\;(0.80,0.10,0.10),\;(0.50,0.20,0.30),\;(0.85,0.05,0.10)\right\}$$

$$q_{L}\in \left\{(0.85,0.15),\;(0.75,0.25),\;(0.90,0.10),\;(0.95,0.05)\right\},$$

a total of twenty reference pairs spanning Th1 fractions from 0.50 to 0.85 and immune fractions from 0.75 to 0.95.

One invariance can be established analytically. On the leukemia axis the divergence

$$D_{L}\left(p^{L}\;∥\;q_{L}\left(\vartheta \right)\right)=p_{11}^{L}\ln\frac{p_{11}^{L}}{\vartheta }+p_{9}^{L}\ln\frac{p_{9}^{L}}{1-\vartheta }$$

is, for each fixed *t*, a strictly decreasing function of the immune fraction $p_{11}^{L}$ on the range $p_{11}^{L}<\vartheta$. Consequently any reference whose immune fraction exceeds those attained along the simulated trajectories ranks the strategies in the same order, and only a reference representing an *unhealthy* target could reverse it. The numerical sweep confirms that this stability extends to the combined divergence. Table 7 reports the minimum combined therapeutic divergence attained by each dosing strategy under all twenty reference pairs.

Three conclusions follow, and we state all three, including the one that does not favor the optimized schedule.

First, the *ordering* of the strategies with respect to the minimum combined divergence is preserved in all twenty reference pairs: the optimal control attains the lowest minimum, followed by the exposure-matched constant dose, the low constant dose, and finally the untreated case. The margin between the two exposure-matched strategies is modest, ranging from 1.3% to 7.4%, but its sign never changes.

Second, the *timing* advantage is both larger and equally stable. The optimal schedule reaches its minimum divergence between 1.2 and 1.5 days earlier than the exposure-matched constant dose in every reference pair, while the untreated and low-dose trajectories are in most cases still decreasing at $t_{f}$ and therefore attain no interior minimum at all. This is consistent with the population-level milestones reported in Table 1 and reinforces the interpretation that the benefit of adaptive scheduling in this model is principally one of rate of response at fixed cumulative exposure.

Third, the same sweep evaluated at the *terminal* time reverses the comparison between the two exposure-matched arms: $D_{\mathrm{tot}}\left(t_{f}\right)$ is higher under optimal control than under the exposure-matched constant dose in all twenty reference pairs. As discussed above, this reflects the withdrawal of the optimized infusion as $\lambda_{7}$ approaches its zero terminal value, after which the leukemic population begins to recover, whereas the constant infusion continues to act until $t_{f}$. The effect is therefore a property of the free terminal state in Formulation (3) rather than of the reference profiles, and it is as robust as the minimum-divergence ordering.

Finally, the absolute magnitudes are strongly reference-dependent: the minimum divergence under optimal control ranges from 0.085 to 0.452 across the twenty pairs, a variation of more than a factor of five. The entropy and divergence values reported in this paper should accordingly be read comparatively, as a means of ranking strategies on a common scale, and not as absolute measures of immunological or disease states.

### 6.6. Entropy of the Helper T-Cell Repertoire

The relative entropy $D_{A}$ measures distance from a prescribed target, whereas the Shannon entropy $S_{A}$ measures how evenly the helper compartment is distributed, independently of any target. The two are complementary, and reading them together resolves a feature of Figure 7 that $D_{A}$ alone leaves ambiguous.

**Figure 8:**
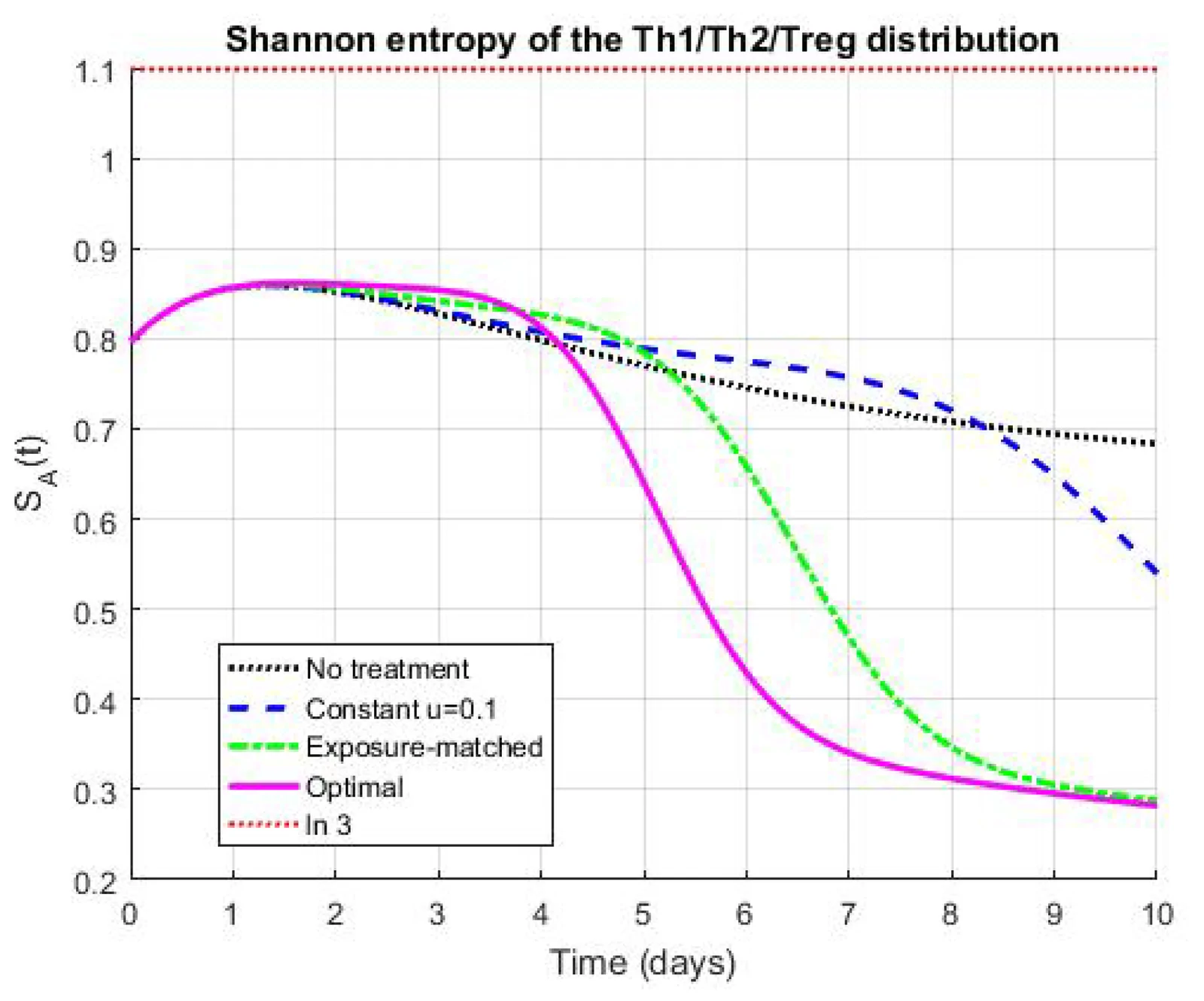
Shannon entropy $S_{A}\left(t\right)$ of the normalized Th1/Th2/Treg distribution under the four dosing strategies. The upper dotted line marks the maximum ln3≈1.099 attained by the uniform distribution.

Figure 8 shows $S_{A}\left(t\right)$. All four strategies begin near 0.80 and rise slightly to approximately 0.86 by day 1.3, as the initially Th2-leaning compartment becomes more evenly mixed. From day 4 the optimal control drives a steep decrease, reaching approximately 0.29 at $t_{f}$; the exposure-matched dose follows the same descent about a day later and reaches a comparable terminal value, while the low constant dose declines only to 0.54 and the untreated case to 0.68.

The decrease is a loss of diversity, and in this setting it is therapeutically desirable: it reflects a directed concentration of the helper compartment on the protective Th1 subset rather than an unstructured collapse. This is precisely why $S_{A}$ is not by itself an adequate diagnostic and must be read alongside $D_{A}$; entropy registers the concentration but not its direction.

The comparison with the reference profile is also informative. The entropy of $q_{A}=\left(0.70,0.10,0.20\right)$ is

$$S\left(q_{A}\right)=-\sum_{i}q_{A,i}\ln q_{A,i}\approx 0.802,$$

so the treated trajectories terminate at an entropy well *below* that of the target profile. The helper distribution under effective treatment is therefore more strongly Th1-concentrated than $q_{A}$ prescribes, which explains why $D_{A}$ plateaus near 0.23 rather than approaching zero in Figure 7: the trajectories do not stop at the reference but pass beyond it. Whether such an overshoot is desirable is a question the present model cannot settle, since $q_{A}$ encodes a qualitative immunological target rather than a calibrated one; we record it as an observation that a patient-calibrated reference profile would need to address.

### 6.7. Divergence-Driven Optimal Control

The diagnostics of Section 6.4 summarize the therapeutic goal in a single dimensionless quantity, which raises the question of whether that quantity could serve as the objective itself, and whether doing so would produce a materially different dosing schedule. We therefore solved a second optimal control problem in which the combined divergence replaces the population-based integrand.

#### 6.7.1. Formulation

Keeping the maximization convention of Section 4, we consider

(21) $$J_{\mathrm{IT}}\left(u\right)=-\int_{0}^{t_{f}}\left[D_{A}\left(t\right)+D_{L}\left(t\right)+A_{5}\;u^{2}\left(t\right)\right]\;dt,\;J_{\mathrm{IT}}\left(u_{\mathrm{IT}}^{*}\right)=\max_{u\in U}J_{\mathrm{IT}}\left(u\right),$$

subject to the same delayed dynamics (Equation (1)) and the same admissible set. Existence follows from Theorem 1 without modification, since the proof uses only that *F* is affine in *u* and that the integrand is concave in *u* and continuous in the state.

#### 6.7.2. Adjoint System

The optimality system changes only in its state-dependent forcing terms. The required gradients are available in closed form.

**Lemma 1** (Gradients of the axis divergences)**.** *Let $\Sigma_{A}:=x_{2}+x_{3}+x_{4}$ and $\Sigma_{L}:=x_{9}+x_{11}$. Then for $k\in J_{A}$ and $\ell \in J_{L}$,*

$$\frac{\partial D_{A}}{\partial x_{k}}=\frac{1}{\Sigma_{A}}\left[\ln\frac{p_{k}^{A}}{q_{A,k}}-D_{A}\right],\;\frac{\partial D_{L}}{\partial x_{\ell }}=\frac{1}{\Sigma_{L}}\left[\ln\frac{p_{\ell }^{L}}{q_{L,\ell }}-D_{L}\right],$$

*and the derivatives with respect to the remaining state variables vanish.*

**Proof.**  Since $p_{i}^{A}=x_{i}/\Sigma_{A}$ we have $\partial p_{i}^{A}/\partial x_{k}=\left(\delta_{ik}-p_{i}^{A}\right)/\Sigma_{A}$, whence

$$\frac{\partial D_{A}}{\partial x_{k}}=\sum_{i\in J_{A}}\frac{\partial p_{i}^{A}}{\partial x_{k}}\left[\ln\frac{p_{i}^{A}}{q_{A,i}}+1\right]=\frac{1}{\Sigma_{A}}\left[\ln\frac{p_{k}^{A}}{q_{A,k}}+1-\left(D_{A}+1\right)\right],$$

the bracketed sum being $D_{A}+1$ because $\sum_{i}p_{i}^{A}=1$. The statement for $D_{L}$ is identical. □

Accordingly, the adjoint equations of Section 5.1 apply verbatim with the constant cost terms replaced as follows:

$$\begin{array}{ccc} \mathrm{in}\;{\dot{\lambda }}_{2}:\;-A_{2}\;\mapsto \;\frac{\partial D_{A}}{\partial x_{2}}, & \mathrm{in}\;{\dot{\lambda }}_{3}:\;+A_{3}\;\mapsto \;\frac{\partial D_{A}}{\partial x_{3}}, & \mathrm{in}\;{\dot{\lambda }}_{4}:\;\;\;0\;\mapsto \;\frac{\partial D_{A}}{\partial x_{4}},\\ \mathrm{in}\;{\dot{\lambda }}_{9}:\;+A_{4}\;\mapsto \;\frac{\partial D_{L}}{\partial x_{9}}, & \mathrm{in}\;{\dot{\lambda }}_{11}:\;-A_{1}\;\mapsto \;\frac{\partial D_{L}}{\partial x_{11}}. & \end{array}$$

Two features deserve note. First, the regulatory compartment $x_{4}$ now enters the optimization: it appears in the allergy-axis distribution but not in Equation (3), so the divergence-based formulation couples Treg dynamics into the control problem where the population-based one did not. Second, since $\partial H_{\mathrm{IT}}/\partial u=\lambda_{7}-2A_{5}u$ exactly as before, the control characterization (Equation (18)) and the numerical scheme of Section 5.4 carry over unchanged.

**Remark 5** (Numerical safeguard)**.** *The gradients of Lemma 1 are singular where a component of $p^{A}$ or $p^{L}$ vanishes. The helper fractions remain bounded away from zero in all simulations, but the leukemic fraction $p_{9}^{L}$ approaches zero under effective treatment. The logarithms were therefore evaluated using fractions floored at $\epsilon_{p}=10^{-10}$ and renormalized; the computed schedule was verified to be insensitive to this choice.*

#### 6.7.3. Comparison of the Two Schedules

**Figure 9:**
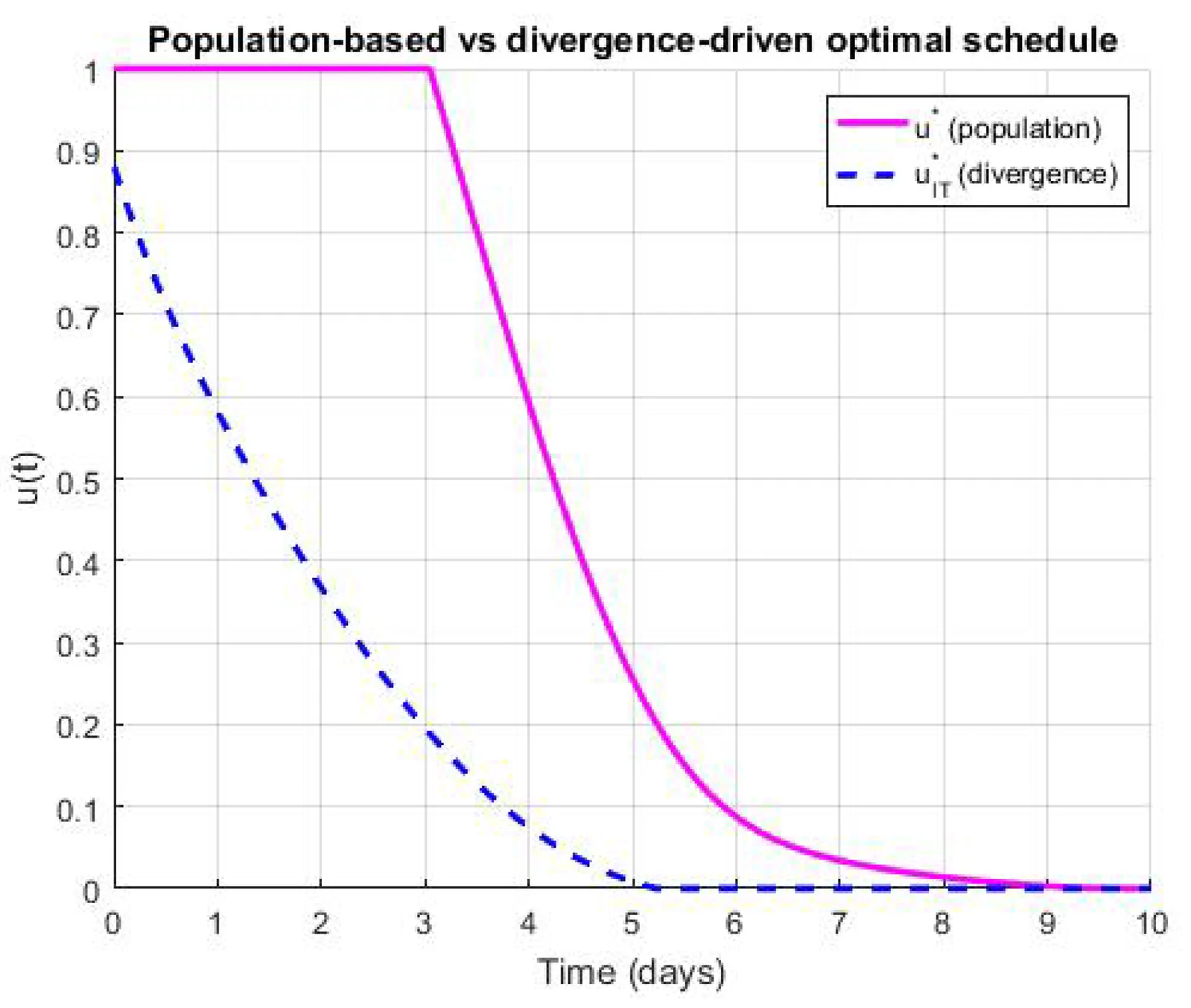
Optimal schedules obtained from the population-based objective (Equation (3)) and from the divergence-based objective (Equation (21)).

Figure 9 compares the two schedules, and the difference is substantial. The divergence-driven control $u_{\mathrm{IT}}^{*}$ never saturates: it begins at approximately 0.87 and decreases smoothly to zero by day 5.5, whereas $u^{*}$ holds the maximal dose for three days before tapering. The cumulative exposures differ accordingly,

$$E\left(u_{\mathrm{IT}}^{*}\right)=1.636\;\mathrm{against}\;E\left(u^{*}\right)=4.460,$$

so the divergence-driven schedule administers 37% of the drug. The resulting terminal leukemic burdens are $x_{9}\left(t_{f}\right)=0.106$ and 0.027 respectively—reductions of 93.2% and 98.3% relative to the untreated terminal value of 1.560.

This is a genuine therapeutic trade-off rather than a numerical artefact, and Proposition 4 explains it. The divergence $D_{L}$ depends on the leukemic population only through the fraction $p_{9}^{L}=x_{9}/\left(x_{9}+x_{11}\right)$, so once that fraction is small the objective is nearly insensitive to further absolute reduction, and the quadratic exposure penalty dominates. The population-based objective, by contrast, contains $x_{9}$ linearly and continues to reward absolute reduction until the terminal time. The information-theoretic formulation therefore prescribes a markedly gentler schedule that forgoes the last five percentage points of leukemic reduction in exchange for a 63% reduction in cumulative drug exposure.

#### 6.7.4. Relation Between the Two Formulations

**Proposition 4** (Scale invariance of the divergence-based objective)**.** *Let $\sigma_{A},\sigma_{L}>0$ and consider the rescaling $x_{i}\mapsto \sigma_{A}x_{i}$ for $i\in J_{A}$ and $x_{i}\mapsto \sigma_{L}x_{i}$ for $i\in J_{L}$. Then $S_{A}$, $D_{A}$, $D_{L}$ and $D_{\mathrm{tot}}$ are unchanged, and hence so is $J_{\mathrm{IT}}$. By contrast J is not invariant, transforming as*

$$J\;⟼\;\int_{0}^{t_{f}}\left[\sigma_{A}\left(A_{2}x_{2}-A_{3}x_{3}\right)+\sigma_{L}\left(A_{1}x_{11}-A_{4}x_{9}\right)-A_{5}u^{2}\right]dt,$$

*so that a change of measurement units acts on J exactly as a reweighting of $A_{1},\ldots ,A_{4}$.*

**Proof.**  $p^{A}$ and $p^{L}$ are normalized fractions, hence invariant under a common positive scaling of the populations entering each axis, and $S_{A}$, $D_{A}$, $D_{L}$ depend on the state only through them. The transformation of *J* follows from the linearity of its state-dependent part. □

**Remark** **6.** *Proposition 4 identifies the practical difference between the two formulations. In Equation *(3)*, the choice of weights cannot be separated from the choice of units in which the compartments are measured, so a weighting that is reasonable under one normalization need not be under another; this is one reason the sensitivity study of Section 6.2 is required. Formulation *(21)* removes that ambiguity at the cost of discarding information about absolute magnitude: it is by construction blind to a proportional reduction in an entire axis. Neither formulation dominates the other. Which is preferable depends on whether the clinical objective is understood as minimizing absolute residual disease or as restoring a favorable immune–leukemic balance at the least cumulative exposure, and the two schedules computed here quantify what that choice costs in each currency.*

### 6.8. Model Assumptions and Limitations

The proposed model is based on several simplifying assumptions that should be considered when interpreting the results. Nonlinear interactions among Th1, Th2, and regulatory T cells are represented through Hill-type suppression functions [9], which capture the principal mechanisms of immune cross-regulation but do not account for stochastic fluctuations that may arise in real immune responses. Three fixed delays, $\tau_{1}$, $\tau_{2}$ and $\tau_{3}$, describe antigen presentation, cytokine production, and leukemic cell-cycle progression; these correspond to average physiological timescales and do not reflect patient-specific variability. Cytokine activity is aggregated into a single compartment, and the immune effector population combines cytotoxic T lymphocytes and natural killer cells into one variable, which may mask distinct functional roles [28,29]. The chemotherapeutic effect follows the classical log-kill hypothesis [1]; drug resistance, pharmacogenomic variability, and individualized pharmacokinetics are not incorporated. Accordingly, the numerical simulations should be interpreted as exploratory and hypothesis-generating rather than as direct clinical predictions.

A related observation follows from the sensitivity study. The half-saturation constant θ of the leukemic self-renewal flux (Equation (2)) has a normalized sensitivity index of order $10^{-4}$ on every outcome, because θ exceeds the leukemic burden attained in the simulations by nearly two orders of magnitude. The saturating nonlinearity is therefore inactive in the present regime, and the leukemic equation operates in its effectively quadratic range, with $\Psi \left(x_{9}\right)\approx \beta_{L}x_{9}^{2}$ throughout. Two consequences should be recorded. The results reported here do not probe the saturation of leukemic self-renewal at high density, so a parameter regime in which that mechanism is active would be required to test it; and, over the range of burdens simulated, the per-cell renewal rate $\Psi \left(x_{9}\right)/x_{9}$ increases with leukemic density rather than decreasing, which departs from the density-limiting form of [19] and should be borne in mind when comparing the present model with that literature.

A further limitation concerns the immune–leukemic coupling. In the parameter regime of Table 8 the interaction coefficients $c_{1}$, $c_{2}$ and ρ are several orders of magnitude smaller than the supply and decay rates *s* and *m*, with the consequence that the effector equation is dominated by its intrinsic supply–decay balance and the effector trajectory is nearly identical across all dosing strategies (Figure 5); the sensitivity indices of these coefficients are indistinguishable from zero on every outcome measure. Two implications follow and should be stated plainly. The reduction in leukemic burden reported here is attributable to the direct cytotoxic term rather than to enhanced immune-mediated clearance; and the finding that immune competence is preserved under optimal dosing is, in this regime, a structural feature of the parameterization rather than an independent prediction of the model. A parameter set in which the immune–tumor interaction is dynamically active would be required to test whether adaptive dosing additionally recruits immune-mediated clearance, and we regard this as the most informative direction for calibration against clinical data.

A tension between the optimized schedule and a clinical consideration raised in the introduction should also be acknowledged. The computed control administers the maximal admissible dose for approximately the first three days, and the leukemic population falls by half within 2.7 days. Rapid destruction of a large leukemic burden is precisely the circumstance associated with tumor lysis syndrome, a complication the present objective functional does not represent. A formulation intended for clinical interpretation would require either a state constraint bounding the rate of leukemic decline or an explicit term penalizing it; the schedules computed here should therefore be read as characterizing the efficacy–exposure trade-off in isolation, and not as directly implementable protocols.

The information-theoretic quantities introduced in Section 6.4 depend on prescribed reference distributions $q_{A}$ and $q_{L}$, which reflect qualitative immunological targets rather than patient-calibrated profiles. This affects both roles in which they are used. As diagnostics, the ordering of dosing strategies is preserved across all twenty alternative reference pairs tested in Section 6.5, but the absolute divergence values vary by more than a factor of five and must be read comparatively. As an objective, in Section 6.7, the reference profiles become design parameters of the control problem, so that the schedule $u_{\mathrm{IT}}^{*}$ inherits whatever clinical judgement is embedded in them; calibrating these profiles against patient data is consequently a prerequisite for any translational use of the divergence-driven formulation, and a more demanding requirement than for the diagnostic use.

From a practical perspective, implementation of adaptive chemotherapy protocols guided by immune dynamics would require repeated monitoring of relevant biomarkers, including cytokine assays, flow cytometry measurements, and immune profiling. Such requirements may increase logistical and financial demands, particularly in resource-limited settings, though continued advances in diagnostic technologies may improve their feasibility.

Finally, while the present study compares the optimized schedule against untreated, low-dose and exposure-matched constant protocols, and against the divergence-driven schedule of Section 6.7, systematic comparison against alternative optimal control formulations in oncology—objective functionals with terminal cost, $L^{1}$ penalties on the control inducing genuinely bang–bang schedules, or explicit state constraints representing toxicity thresholds—was not undertaken and remains an important topic for future investigation. The first of these is of particular interest here, since a terminal cost on the leukemic population would remove the late rebound documented in Section 6.5. Future extensions may also include patient-specific parameter estimation, multiple cytokine subclasses, stochastic immune fluctuations, drug-resistance mechanisms, and calibration using clinical datasets.

## 7. Conclusions and Recommendations

The numerical simulations indicate that the proposed optimal control strategy improves therapeutic outcomes within the model when compared with the absence of treatment, with a low constant dose, and—most informatively—with a constant infusion delivering the same cumulative drug exposure. Because the last of these holds the total drug administered fixed at E=4.4596, the differences between it and the optimized schedule are attributable to the temporal distribution of the dose alone. On that comparison the optimal schedule improves the objective functional by 40.5% (J=24.15 against 17.18) and reaches every therapeutic milestone between 0.9 and 1.6 days earlier, while attaining a terminal leukemic burden lower by a factor of 2.25. The benefit of adaptive scheduling in this model is therefore principally one of *rate of response at fixed drug exposure* rather than of eventual position: given the same total drug, a constant infusion reaches a broadly comparable configuration, only later.

The principal observations may be summarized as follows:

- **Effector Th1 cells ($x_{2}$).** Under the optimized strategy the Th1 population expands rapidly from approximately day 4, overtaking Th2 at day 3.74 and reaching 7.011 by the end of the horizon. The exposure-matched constant dose attains a comparable terminal level of 6.803 but crosses over at day 4.61, while the low constant dose produces only a limited late increase to 2.073 and the untreated case remains at 0.286. The distinguishing feature is thus the timing of the expansion rather than its eventual magnitude.
- **Suppressor Th2 cells (*x*_3_).** In absolute terms the Th2 population rises under all scenarios and is in fact slightly highest under optimal control. The therapeutic benefit therefore lies in the Th1/Th2 *ratio* rather than the absolute Th2 level. Within the Th1/Th2 framework adopted here, this shift in proportion is the mechanism by which the model represents a reduced risk of allergic hypersensitivity; the model does not represent hypersensitivity events directly, and the association should be read as a modeling assumption inherited from [9,10] rather than a validated clinical endpoint. The sensitivity study further shows that the Th2 penalty $A_{3}$ has almost no influence on the computed schedule, since Th2 varies by less than 15% across all strategies.
- **Leukemic cells (*x*_9_).** The leukemic burden falls to 0.0267 under the optimal schedule and 0.0601 under the exposure-matched constant dose, against 0.4474 for the low constant dose and continued slight progression when untreated. In the parameter regime considered this reduction is driven by the direct cytotoxic term: the interaction coefficients $c_{1}$, $c_{2}$ and ρ have sensitivity indices indistinguishable from zero, so the model does not support an additional claim of enhanced immune-mediated clearance.
- **Immune effector cells (*x*_11_).** The effector population grows almost identically under all four scenarios, indicating that within the present model it is governed by its intrinsic supply–decay dynamics and is not measurably suppressed by chemotherapy over this horizon. The optimal strategy therefore achieves substantial leukemic reduction without compromising immune competence, though this should be understood as a structural feature of the parameterization rather than an independent prediction.
- **Information-theoretic diagnostics.** The relative-entropy diagnostics on the allergy and leukemia axes, together with their combined divergence, characterize both treatment goals in a single scale-free framework. Under optimal control both axes approach their healthy targets fastest, and the minimum combined divergence is both lowest (0.194 against 0.203) and earliest (day 5.82 against day 7.19); this ordering is preserved across all twenty alternative reference profiles tested. At the terminal time, however, the comparison reverses—$D_{\mathrm{tot}}\left(t_{f}\right)=0.333$ under optimal control against 0.292 under the exposure-matched dose—because the optimized infusion is withdrawn as the costate approaches its zero terminal value while the constant infusion continues to act. This reversal is equally robust across reference profiles and is a property of the free terminal state rather than of the diagnostic.
- **Divergence-driven control.** When the combined divergence is taken as the running cost of a second optimal control problem, the resulting schedule differs materially: it never saturates, administers only 37% of the drug (E=1.636 against 4.460), and still achieves a 93.2% leukemic reduction against 98.3% for the population-based formulation. Because the divergence depends on the leukemic population only through a normalized fraction, it becomes insensitive to further absolute reduction once that fraction is small. The two formulations of the therapeutic goal therefore do not induce the same optimal policy, and the comparison quantifies what the choice between them costs in each currency.

Overall, these findings suggest that adaptive, immune-aware chemotherapy scheduling may accelerate disease control and the establishment of a Th1-favorable helper balance at fixed cumulative drug exposure, and that information-theoretic objectives offer a scale-free alternative formulation of the therapeutic goal that prescribes substantially gentler dosing. The numerical results are supported by documented convergence of the optimality system—geometric at a rate of approximately 0.55 per iteration, with the iteration count independent of the discretization—and by grid independence to six significant figures. A one-at-a-time sensitivity study over kinetic parameters, delays, initial conditions and objective weights leaves the qualitative structure of the optimal schedule and the ordering of the four strategies unchanged; the drug-induced leukemic death rate $\mu_{L}$ is the only parameter whose relative perturbation is amplified, with a normalized sensitivity index of −4.53 on the terminal leukemic burden.

The limitations set out in Section 6.8 should nevertheless be borne in mind, in particular that the immune–leukemic coupling is dynamically inactive in the present parameter regime, that the schedules computed here administer maximal dose over an initial phase without representing tumor lysis risk, and that the conclusions rest on simulation without direct clinical validation.

**Recommendations.** Future therapeutic protocols for CLL may benefit from incorporating immune monitoring to guide individualized chemotherapy dosing. In particular, the Th1/Th2 ratio—and, more generally, information-theoretic measures such as the entropy and relative entropy of the helper T-cell and immune/leukemic distributions—could serve as useful biomarkers for balancing treatment efficacy and safety. Three directions follow most directly from the present results. First, experimental determination of $\mu_{L}$ would be the single most valuable refinement, since it is the only parameter whose uncertainty is amplified in the outcome. Second, a formulation incorporating either a terminal cost on the leukemic population or a state constraint bounding its rate of decline would address, respectively, the late rebound documented here and the tumor lysis risk associated with an initial maximal-dose phase. Third, calibration of the reference profiles $q_{A}$ and $q_{L}$ against patient data is a prerequisite for any translational use of the divergence-driven formulation, since in that formulation they cease to be diagnostic conventions and become design parameters of the control problem. Further work should also include patient-specific parameter estimation, comparison with alternative optimal control strategies, and extension to combination therapies or multi-drug protocols.

## 8. Parameters

Parameter values used in the simulations are listed below. Values taken directly from the cited source are given as such; values that were calibrated so that the untreated system reproduces the slow progression characteristic of chronic lymphocytic leukemia are marked *calibrated*, with the source that informed the choice given for comparison.

The parameters governing leukemic self-renewal and clearance ($\beta_{L}$, θ, $\gamma_{L}$) were calibrated rather than adopted directly from [19], which reports values for chronic myelogenous leukemia. Adopted directly, those values cause the untreated system to clear the leukemic population within approximately two days, which is inconsistent with the indolent progression characteristic of chronic lymphocytic leukemia. The calibrated values instead reproduce a near-stationary untreated trajectory: over the ten-day horizon the untreated leukemic burden rises from 1.500 to 1.560 (Figure 4), corresponding to an exponential growth rate of $3.9\times 10^{-3}$ day^−1^ and a lymphocyte doubling time of 177 days, or approximately 5.8 months. Lymphocyte doubling time has been established as a prognostic marker in chronic lymphocytic leukemia since [38], and a doubling time below six months is one of the criteria for active disease indicating that treatment is warranted [39]. The calibration therefore places the untreated model in the progressive-disease regime for which the treatment schedules studied here would in practice be considered, rather than being chosen to produce a particular treatment outcome.

The sensitivity study of Section 6.2 indicates that this calibration does not drive the conclusions: the normalized sensitivity indices of $\beta_{L}$, $\gamma_{L}$ and θ on the terminal leukemic burden are 0.32, −0.11 and $10^{-4}$, respectively, so a ±20% uncertainty in any of them leaves the reported comparisons unchanged.

The drug pharmacokinetic parameters ($\gamma_{D}$, $\mu_{D}$, *d*), the helper-cell death rates ($\beta_{2},\beta_{3},\beta_{4}$) and the naive APC supply rate ($a_{APC}$) are likewise calibrated values informed by the cited sources rather than direct adoption of the reported figures. Two half-saturation constants appear in the model with very different magnitudes: a=2 in the drug-elimination and cytotoxic terms, and $c=7.14\times 10^{-7}$ in the immune-mortality term, although both refer to the same drug concentration $x_{7}$. The latter follows the normalization of [37], in which the corresponding parameter is reported on a scale that differs from the one used here; with $c\ll x_{7}$ over almost the whole horizon, the factor $x_{7}/\left(c+x_{7}\right)$ is effectively unity once treatment begins, so the immune-mortality term reduces to $\delta x_{11}$. Since $\delta =1.42\times 10^{-3}$ is two orders of magnitude below the effector supply rate, this term is in any case negligible, as the sensitivity indices of both δ and *c* confirm. 

## Figures and Tables

**Table 1:** Quantitative comparison of the four dosing strategies. E(u) is the cumulative normalized drug exposure; $t_{\mathrm{cross}}$ is the first time at which $x_{2}$ exceeds $x_{3}$; $t_{50}$ and $t_{90}$ are the times at which the leukemic burden falls below 0.75 and 0.15, corresponding to reductions of 50% and 90% from the initial value; $D_{\mathrm{tot}}^{\min}$ is the minimum of the combined therapeutic divergence and $t_{\min}$ the time at which it is attained; *J* is the value of the objective functional (Equation (3)) at $A_{1}=\ldots =A_{5}=1$.

Strategy	E(u)	$x_{9}\left(t_{f}\right)$	$x_{2}\left(t_{f}\right)$	$x_{3}\left(t_{f}\right)$	$x_{2}/x_{3}$	$t_{\mathrm{cross}}$	$t_{50}$	$t_{90}$	$D_{\mathrm{tot}}\left(t_{f}\right)$	$D_{\mathrm{tot}}^{\min}$ ($t_{\min}$)	*J*
No treatment	0	1.560	0.286	0.527	0.54	—	—	—	1.292	1.292 (10.00)	−8.0289
Constant u=0.1	1.000	0.4474	2.073	0.577	3.60	6.96	7.36	—	0.2661	0.2661 (10.00)	−0.6452
Exposure-matched $\bar{u}$	4.4596	0.0601	6.803	0.598	11.37	4.61	3.78	8.05	0.2924	0.2032 (7.19)	17.1836
Optimal $u^{*}$	4.4596	0.0267	7.011	0.599	11.71	3.74	2.74	6.43	0.3327	**0.1942** (5.82)	**24.1465**

Bold marks the best value attained across the four strategies.

**Table 2:** Normalized sensitivity indices under ±20% perturbation of the kinetic parameters, with absolute shifts in $t_{50}$ and $t_{90}$ in days. Parameters for which all indices vanish to four decimal places are grouped in the final row.

Parameter	$S^{x_{9}\left(t_{f}\right)}$	$S^{E(u^{*})}$	$S^{D_{\mathrm{tot}}^{\min}}$	$\Delta t_{50}$ (d)	$\Delta t_{90}$ (d)
$\mu_{L}$	−4.5263	−0.0239	−0.0839	−0.380	−1.016
$\beta_{L}$	0.3164	0.0030	0.0081	0.036	0.105
$\gamma_{L}$	−0.1059	−0.0010	−0.0022	−0.015	−0.028
$\gamma_{D}$	0.0775	0.0231	0.0019	0.003	0.012
*v*	0.0654	−0.0867	−0.0980	0.000	0.006
$\eta_{r}$	−0.0009	0.0006	0.2122	0.000	0.000
κ	0.0008	−0.0006	−0.0470	0.000	0.000
ϕ	0.0015	−0.0008	0.0256	0.000	0.000
*s*	≈0	≈0	−0.0210	0.000	0.000
$\mu_{D}$	0.0033	0.0015	0.0002	0.000	0.000
θ	0.0001	0.0000	0.0000	0.000	0.000
$\mu_{r}$	−0.0009	0.0012	0.0002	0.000	0.000
$c_{1},\;c_{2},\;\rho ,\;m,\;\delta$	≈0	≈0	≤0.0002	0.000	0.000

Bold marks the only index whose magnitude exceeds unity, indicating amplification of the relative perturbation.

**Table 3:** Sensitivity to the three delays under ±20% perturbation.

Delay	$S^{x_{9}\left(t_{f}\right)}$	$S^{E(u^{*})}$	$S^{D_{\mathrm{tot}}^{\min}}$	$\Delta t_{50}$ (d)	$\Delta t_{90}$ (d)
$\tau_{1}$	0.0056	−0.0061	0.0010	0.000	0.000
$\tau_{2}$	0.0000	−0.0000	−0.0008	0.000	0.000
$\tau_{3}$	0.4500	0.0030	0.0086	−0.001	0.121

**Table 4:** Dependence on the initial leukemic burden $x_{9}\left(0\right)$. The switching time is the instant at which the optimal control leaves saturation. Note that $t_{50}$ and $t_{90}$ are times to reach the *absolute* levels 0.75 and 0.15, not relative reductions, so the first row reflects an initial state already below the first threshold.

$x_{9}\left(0\right)$	$x_{9}\left(t_{f}\right)$	$E(u^{*})$	$D_{\mathrm{tot}}^{\min}$	$t_{50}$ (d)	Switching Time (d)
0.50	0.00719	4.4119	0.2366	0	2.97
1.00	0.01603	4.4345	0.2001	1.538	3.01
1.50	0.02670	4.4596	0.1942	2.734	3.05
2.00	0.03936	4.4872	0.2014	3.554	3.09
3.00	0.07148	4.5492	0.2149	4.798	3.18

**Table 5:** Effect of the objective weights. All weights other than the one listed are held at unity. A switching time of zero indicates that the control never saturates.

Weight	Value	$x_{9}\left(t_{f}\right)$	$E(u^{*})$	$D_{\mathrm{tot}}^{\min}$	$t_{50}$ (d)	Switching Time (d)
$A_{5}$	0.5	0.02385	5.0461	0.19367	2.734	4.00
	1	0.02670	4.4596	0.19422	2.734	3.05
	2	0.03243	3.7704	0.19580	2.748	1.50
	5	0.05465	2.7865	0.20079	3.130	0
$A_{2}$	0.5	0.03170	3.8350	0.19558	2.742	1.76
	1	0.02670	4.4596	0.19422	2.734	3.05
	2	0.02395	5.0184	0.19368	2.734	3.98
	5	0.02183	5.6759	0.19346	2.734	4.82
$A_{4}$	0.5	0.02687	4.4297	0.19426	2.734	3.00
	1	0.02670	4.4596	0.19422	2.734	3.05
	2	0.02638	4.5164	0.19416	2.734	3.14
	5	0.02560	4.6675	0.19401	2.734	3.36
$A_{3}$	0.5	0.02668	4.4616	0.19422	2.734	3.05
	1	0.02670	4.4596	0.19422	2.734	3.05
	2	0.02672	4.4556	0.19423	2.734	3.04
	5	0.02680	4.4433	0.19425	2.734	3.00

**Table 6:** Grid independence of the computed optimal control. Differences are measured against the next coarser grid on the interior interval [0.5,9.5], after linear interpolation onto a common mesh.

*N*	*h*	Iterations	$x_{9}\left(t_{f}\right)$	$E(u^{*})$	$∥u_{h}-u_{h/2}{∥}_{\infty }$
2500	$4\times 10^{-3}$	23	0.0266961	4.45963	—
5000	$2\times 10^{-3}$	23	0.0266962	4.45962	$1.43\times 10^{-4}$
10,000	$1\times 10^{-3}$	23	0.0266962	4.45962	$2.71\times 10^{-7}$

**Table 7:** Minimum combined therapeutic divergence $D_{\mathrm{tot}}^{\min}$ over $\left[0,t_{f}\right]$ for each dosing strategy under twenty reference pairs; the time at which the minimum is attained is given in parentheses for the two exposure-matched strategies. The optimal schedule attains the lowest minimum in all twenty cases. Bold marks the lowest minimum divergence in each row, attained by the optimal schedule in all twenty cases.

$q_{A}$	$q_{L}$	No Treatment	Constant u=0.1	Exposure-Matched	Optimal	Ordering
(0.70,0.10,0.20)	(0.85,0.15)	1.292	0.2661	0.2032 (7.2)	0.1942 (5.8)	preserved
(0.70,0.10,0.20)	(0.75,0.25)	1.092	0.2434	0.2213 (6.8)	0.2100 (5.5)	preserved
(0.70,0.10,0.20)	(0.90,0.10)	1.471	0.3166	0.2107 (7.7)	0.2031 (6.3)	preserved
(0.70,0.10,0.20)	(0.95,0.05)	1.799	0.4363	0.2240 (9.2)	0.2175 (7.7)	preserved
(0.60,0.20,0.20)	(0.85,0.15)	0.8989	0.2360	0.2000 (6.3)	0.1863 (5.1)	preserved
(0.60,0.20,0.20)	(0.75,0.25)	0.6989	0.2057	0.1790 (6.0)	0.1635 (4.8)	preserved
(0.60,0.20,0.20)	(0.90,0.10)	1.078	0.2864	0.2371 (6.6)	0.2253 (5.3)	preserved
(0.60,0.20,0.20)	(0.95,0.05)	1.406	0.4061	0.3040 (8.6)	0.2932 (7.2)	preserved
(0.80,0.10,0.10)	(0.85,0.15)	1.250	0.1636	0.0914 (7.3)	0.0849 (6.0)	preserved
(0.80,0.10,0.10)	(0.75,0.25)	1.049	0.1408	0.1158 (6.9)	0.1078 (5.6)	preserved
(0.80,0.10,0.10)	(0.90,0.10)	1.429	0.2140	0.0942 (7.9)	0.0886 (6.5)	preserved
(0.80,0.10,0.10)	(0.95,0.05)	1.757	0.3338	0.1028 (9.3)	0.0979 (7.8)	preserved
(0.50,0.20,0.30)	(0.85,0.15)	0.9600	0.3757	0.3310 (6.1)	0.3129 (4.9)	preserved
(0.50,0.20,0.30)	(0.75,0.25)	0.7599	0.3349	0.2982 (5.8)	0.2780 (4.6)	preserved
(0.50,0.20,0.30)	(0.90,0.10)	1.139	0.4277	0.3763 (6.4)	0.3605 (5.1)	preserved
(0.50,0.20,0.30)	(0.95,0.05)	1.467	0.5474	0.4630 (7.1)	0.4517 (5.7)	preserved
(0.85,0.05,0.10)	(0.85,0.15)	1.675	0.2668	0.1188 (7.9)	0.1155 (6.5)	preserved
(0.85,0.05,0.10)	(0.75,0.25)	1.475	0.2440	0.1642 (7.4)	0.1591 (6.0)	preserved
(0.85,0.05,0.10)	(0.90,0.10)	1.854	0.3172	0.1083 (8.4)	0.1060 (6.9)	preserved
(0.85,0.05,0.10)	(0.95,0.05)	2.182	0.4370	0.1055 (9.6)	0.1042 (8.1)	preserved

**Table 8:** Model parameters used in the simulations. Values taken directly from the cited source are given as such; values marked calibrated were adjusted so that the untreated system reproduces the slow progression characteristic of chronic lymphocytic leukemia, with the source that informed the choice given for comparison.

The production rate of naive CD4+ cells. [30]	*α*	0.1
The strength of suppression rate of Th1 by Th2 [9]	$\mu_{2}$	0.1
The strength of suppression of Th2 by Th1 [9]	$\mu_{1}$	0.2
The strength of suppression rate by Treg [9]	$\mu_{r}$	0.25
The differences in the autocrine action of the three subsets [9]	ϕ	0.05
The differences in the autocrine action of the three subsets [9]	κ	0.1
The death rate of immature APCs [30]	$\gamma_{11}$	0.08 day^−1^
The death rate of mature APCs [30]	$\gamma_{12}$	0.8 day^−1^
The death rate of Naive T cells [30]	$\beta_{1}$	0.03 day^−1^
The death rate of $T_{1}$ cells, calibrated; cf. [31]	$\beta_{2}$	$4.16*10^{-5}$ day^−1^
The death rate of $T_{2}$ cells, calibrated; cf. [31]	$\beta_{3}$	$4.16*10^{-5}$ day^−1^
The death rate of $T_{reg}$ cells, calibrated; cf. [31]	$\beta_{4}$	$4.16*10^{-5}$ day^−1^
The proliferation rate of stimulated T-cells [9]	*v*	4
Natural decay of induced cytokine during chemotherapy [29]	$\gamma_{2}$	0.4152 day^−1^
Inhibition rate of Treg cells by the induced cytokines [32]	$\eta_{r}$	0.4 day^−1^
First time delay [33]	$\tau_{1}$	0.0794 day
Second time delay [29]	$\tau_{2}$	0.25 day
Third time delay [34]	$\tau_{3}$	2.8 day
The production of induced cytokines by other cells [35]	$k_{1}$	1
The supply rate of naive APCs, calibrated; cf. [30]	$a_{APC}$	2
Rate of APC activation by the antigen [36]	$\beta_{0}$	0.01
Rate of APC inhibition by regulatory T cells [36]	$\mu_{0}$	$10^{-2}$
Chemical deactivation rate of drug, calibrated; cf. [1]	$\gamma_{D}$	0.0192 day^−1^
Deactivation rate of drug due to killing of tumor cells, calibrated; cf. [1]	$\mu_{D}$	$7.48*10^{-3}$ day^−1^
Drug dose that produces 50% maximum effect [1]	*a*	$2*10^{3}$ mL = 2 in microliter
Natural death rate of leukemic cells, calibrated; cf. [19]	$\gamma_{L}$	0.01 day^−1^
Death rate resulting from the action of drug on cancer cells estimated from [1]	$\mu_{L}$	0.74 day^−1^
Rate of dissolution of dead tumor cells, calibrated; cf. [1]	*d*	$7.07*10^{-4}$ day^−1^
The supply rate of immune cells [37]	*s*	$7*10^{5}$ cells day^−1^ = 0.1 cells μL^−1^ day^−1^
Natural death rate of immune cells [37]	*m*	$10^{-3}$ day^−1^
Rate of immune cell population activated by the tumor [37]	ρ	$10^{-12}$ day^−1^
Half-saturation in immune activation [37]	γ	$10^{2}$ cells = $1.42*10^{-5}$ cells μL^−1^
The mortality rate of immune cells due to the chemotherapeutic drug [37]	δ	$1.42*10^{-3}$ day^−1^
Half-saturation parameter [37]	*c*	$7.14*10^{-7}$ in microliter
Rate of elimination of tumor cells by immune cells [37]	$c_{1}$	$5*10^{-11}$ μL cells^−1^ day^−1^
Rate of immune cells which directly eliminate tumor cells [37]	$c_{2}$	$10^{-13}$ μL cells^−1^ day^−1^
Component of the rate of self-renewal, calibrated; cf. [19]	$\beta_{L}$	0.01 day^−1^
Component of the rate of self-renewal, calibrated; cf. [19]	θ	100 cells μL^−1^

**Note on calibration.** All concentrations are expressed per microlitre; conversions from absolute cell counts use a total blood volume of $7\times 10^{6}$ μL.

## Data Availability

The MATLAB code implementing the forward–backward sweep and generating all figures and tables reported in this article is available from the corresponding author upon reasonable request. No new data were created.
